# Adaptive Active Illumination Via Translationally and Axially Tunable Cascaded Metasurfaces

**DOI:** 10.1002/advs.78448

**Published:** 2026-10-27

**Authors:** Chi Zhang, Jianing Yang, Lingyun Zhang, Jingyu Bao, Bingbai Li, Qingsong Feng, Rui You, Xiaoli Jing, Fei Xing, Xiaoguang Zhao, Zheng You

**Affiliations:** ^1^ State Key Laboratory of Precision Measurement Technology and Instruments Tsinghua University Beijing China; ^2^ Department of Precision Instrument Tsinghua University Beijing China; ^3^ State Key Laboratory of Intelligent Manufacturing Equipment and Technology School of Mechanical Science and Engineering Huazhong University of Science and Technology Wuhan China; ^4^ Beijing Institute of Tracking and Telecommunications Technology Beijing China; ^5^ Laboratory of Intelligent Microsystems Beijing Information Science and Technology University Beijing China; ^6^ Beijing Advanced Innovation Center for Integrated Circuits Tsinghua University Beijing China

**Keywords:** active illumination, three‐dimensional sensing, tunable metasurfaces, beam shaping

## Abstract

Active three‐dimensional sensing requires accurate light delivery and sufficient return‐signal strength, while complex scenes require a tunable balance between local power concentration and illumination coverage. Collimated beam illumination supports a longer maximum detectable distance by concentrating optical power, whereas expanded beam illumination improves angular sampling efficiency by increasing coverage per projection. Existing active‐illumination approaches typically use fixed illumination patterns or predefined scanning trajectories, making it difficult to balance local power concentration for maximum detectable distance with illumination coverage for sampling efficiency. Here we propose an adaptive active‐illumination method based on translationally and axially tunable cascaded metasurfaces (TATCMs). TATCMs control the beam output angle within the field of view (FoV) through lateral translation and tune illumination coverage through axial‐spacing tuning. At an operating wavelength of 905 nm, the implemented TATCMs demonstrate continuous beam‐output‐angle control across an approximately 70°×70° FoV under a ±300 µm lateral stroke, while increasing the interlayer spacing by 2.64 mm expands collimated beam illumination with a measured divergence of 0.06° to provide continuous coverage across the FoV. Adaptive three‐dimensional reconstruction further demonstrates the potential of TATCMs as a compact planar illumination platform for scene‐adaptive three‐dimensional sensing in autonomous vehicles, unmanned aerial vehicles, and industrial robotics.

## Graphical abstract

Active three‐dimensional sensing faces a fundamental trade‐off between seeing farther and covering more of the scene. Translationally and axially tunable cascaded metasurfaces overcome this limitation by continuously adapting beam direction and illumination coverage. The resulting platform concentrates light where stronger returns are needed and broadens illumination where faster sampling is preferred, enabling compact scene‐adaptive sensing.

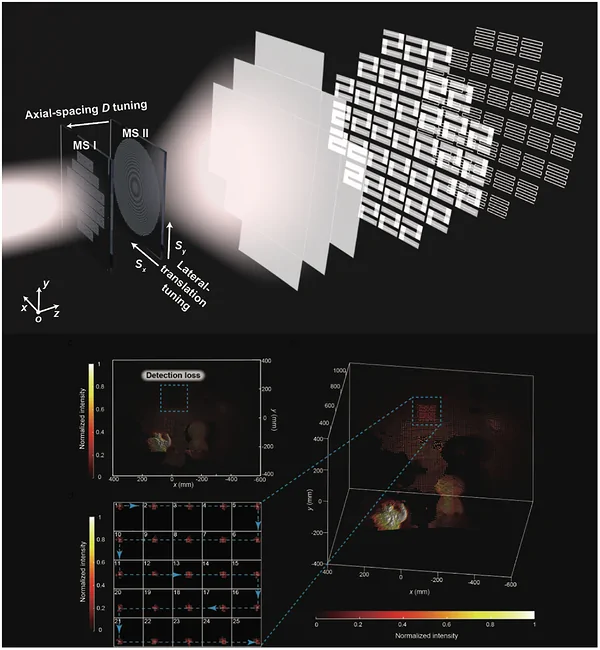

## 1 Introduction

Active three‐dimensional sensing systems project controlled illumination onto a scene and recover depth information from the returned optical signals [1]. The controlled illumination reduces the dependence on ambient light and natural surface textures, enabling reliable recovery of object position and surface geometry [2]. The illumination optics shape the spatial distribution of projected light across the scene and thereby influence spatial sampling, returned signal strength, and depth reconstruction quality. The growing adoption of active three‐dimensional sensing in mobile platforms [3], unmanned aerial systems [4], and embodied intelligent systems [5] further increases the demand for compact and efficient active‐illumination optics [6].

Active illumination in three‐dimensional sensing involves a trade‐off between maximum detectable distance and angular sampling efficiency [5, 7]. Sensing distant or low‐reflectivity targets requires sufficient returned signal strength, and resolving small structures and object boundaries requires precise spatial localization. These requirements favor collimated beam illumination [8], which concentrates optical power within a small angular range and provides well‐localized sampling positions. Continuous surfaces and rapid scene acquisition instead benefit from expanded beam illumination [9], which increases the coverage obtained in each projection and improves angular sampling efficiency. Practical scenes often combine distant or low‐reflectivity targets with continuous surfaces within the same field of view (FoV) [10]. A fixed illumination scheme cannot adjust local power concentration and illumination coverage to these different sensing requirements. Predefined pattern sequences, denser scanning or higher optical power can provide compensation, but they increase system complexity or power consumption. Achieving a tunable balance between local power concentration and illumination coverage with compact active‐illumination optics therefore remains a key challenge [11].

Conventional active‐illumination systems use refractive lenses, mirrors, and diffractive optical elements to shape the projected light, but their fixed optical responses limit dynamic control of the illumination distribution [12]. Spatial light modulators can electronically reconfigure illumination patterns, but their micrometer‐scale pixels limit the spatial phase gradients that can be encoded and lead to increasingly pronounced phase discretization and efficiency loss at large steering angles [13]. A reported LCOS beam‐steering system [14] achieved a one‐dimensional scanning range of ±15.6°, but the first‐order diffraction efficiency into the desired steered beam was only 2.28% at the maximum steering angle, while the required polarization and relay optics further increased the system size. Metasurfaces use subwavelength meta‐atoms to control optical wavefronts with fine spatial resolution, providing compact optical platforms for three‐dimensional sensing, including structured‐light projection [15], binocular depth perception [16], and wide‐angle beam steering [17]. Reconfigurable beam shaping for adaptive active illumination can be achieved through material‐based tuning or mechanical tuning. Material‐based tuning modifies the optical properties of the constituent materials, but trade‐offs among modulation range, active aperture, and driving complexity can restrict its use in active illumination [18, 19]. A recently reported CMOS‐driven liquid‐crystal metasurface [20] can achieve a one‐dimensional beam‐steering range of 24°, but required 2500 independently addressable electrodes, while the steered‐beam diffraction efficiency decreased from approximately 40% to ~18% at the maximum angle.

Mechanical tuning of cascaded and multilayer metasurfaces provides a practical route to large‐range wavefront control at visible and near‐infrared wavelengths because different mechanical degrees of freedom modify distinct interlayer phase relationships [21]. In‐plane translation changes the lateral registration between phase profiles to reconfigure the cascaded phase distribution, as demonstrated in Alvarez focal tuning [22] and beam steering [23, 24]. Axial tuning changes the propagation distance between layers and thereby modifies the interlayer propagation phase, shifting the focal plane along the optical axis [25]. Relative rotation changes the angular registration between phase profiles and is widely used in Moiré metalenses for continuous focal‐position tuning [26, 27, 28]. These studies establish interlayer geometry as a deterministic control variable for wavefront synthesis in planar optics. However, existing mechanically tunable metasurfaces are generally limited to controlling a single optical function. Adaptive control of both illumination position and illumination coverage with mechanically tunable metasurfaces remains unexplored.

We propose adaptive active illumination based on translationally and axially tunable cascaded metasurfaces (TATCMs). TATCMs control the beam output angle within the FoV through lateral translation, while axial‐spacing tuning transforms collimated beam illumination into expanded beam illumination with tunable illumination coverage. In an experimental implementation at the wavelength of 905 nm, a ±300 µm lateral stroke provides continuous beam‐output‐angle control across an approximately 70°×70° FoV, while a 2.64 mm axial‐spacing change transforms collimated beam illumination with a measured divergence of 0.06° into expanded beam illumination. An adaptive three‐dimensional reconstruction demonstration shows that the illumination state can be selected according to scene‐specific sensing requirements, allowing local power concentration to be increased for weak or distant targets and illumination coverage to be expanded for efficient sampling of continuous surfaces. By enabling a tunable balance between maximum detectable distance and angular sampling efficiency in a compact planar architecture, TATCMs provide an adaptive active‐illumination platform for compact three‐dimensional sensing.

## 2 Results

### 2.1 Design of TATCMs for Adaptive Active Illumination

In active three‐dimensional sensing, illumination with a controlled spatial distribution is projected onto a target scene to generate measurable return signals. The projected distribution determines the balance between optical power concentration and spatial coverage. Adaptive active illumination redistributes finite optical power according to sensing requirements in different scene regions. Detecting weak‐return regions and fine details benefits from concentrated projected spots, which enhance returned signals and improve spatial localization. Reconstructing continuous surfaces and extended regions benefits from broader illumination coverage, which improves sampling completeness. To satisfy different active‐illumination requirements, we design TATCMs comprising the first metasurface (MS I) and the second metasurface (MS II) (Figure 1a). A collimated incident beam is first modulated by MS I, propagates across an axial interlayer spacing *D*, and then passes through MS II to form the output beams. The lateral translation of MS II, denoted by ***S*** = (*S_x_*, *S_y_*), controls the beam output angle associated with each MS I subaperture within the FoV. The axial motion of MS I changes *D*, thereby adjusting the illumination coverage of the beam generated by each MS I subaperture.

**FIGURE 1:**
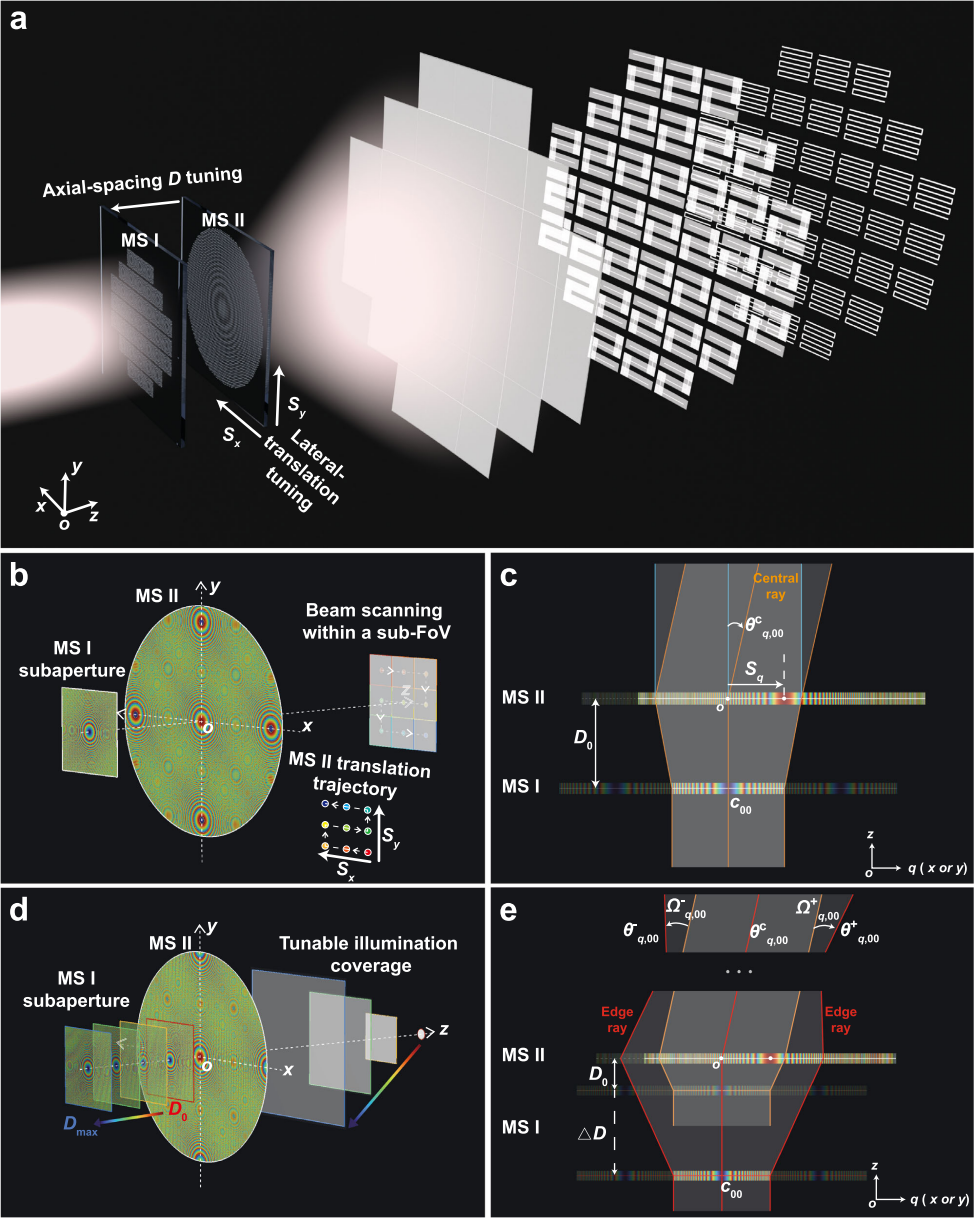
Design principle of translationally and axially tunable cascaded metasurfaces (TATCMs) for adaptive active illumination. (a) Overall schematic of TATCMs for adaptive active illumination. (b) Schematic of beam scanning within a subregion of the field of view (FoV). (c) Cross‐sectional view of the beam output angle at the design spacing *D*_0_. (d) Schematic of axial‐spacing tuning for changing illumination coverage. (e) Cross‐sectional view of illumination coverage generated by axial‐spacing change.

Because the output wavefront is formed on the output plane of MS II, we define the MS II plane as the fixed *x*‐*y* reference plane, with the positive *z* direction pointing from MS I to MS II. When ***S*** = (0, 0), the centers of MS II and MS I are located at (0, 0, 0) and (0, 0, −*D*), respectively, with the positive *z* direction pointing from MS I to MS II. A transverse position on the MS II plane is denoted by ***r***_II_ = (*x*_II_, *y*_II_). MS I contains *N_x_* × *N_y_* subapertures that divide the target FoV into multiple FoV subregions. The center of the (*n*, *m*)‐th subaperture is denoted by ***c*** *_nm_* = (*x_n_*, *y_m_*), where *n* and *m* index the *x* and *y* directions, respectively. For the symmetric design, each MS I subaperture is a square region with side length *L*, and the coordinate domain of the (*n*, *m*)‐th subaperture is *x_n_* − *L*/2 ≤ *x* ≤ *x_n_* + *L*/2 and *y_m_* − *L*/2 ≤ *y* ≤ *y_m_* + *L*/2. After propagation across the interlayer spacing *D*, the wavefront from the (*n*, *m*)‐th MS I subaperture is represented on the MS II plane by the equivalent phase *ϕ*_I→II,_ *_nm_* (***r***_II_; *D*). The phase profile of MS II is denoted by *ϕ*_II_(***r***_II_). When MS II is laterally translated by ***S***, its phase profile becomes *ϕ*_II_(***r***_II_ − ***S***) on the fixed reference plane, and the cascaded output phase for the beam generated by the (*n*, *m*)‐th MS I subaperture is given by

(1) $$\phi_{\mathrm{out},nm}\left(r_{\mathrm{II}};D,S\right)=\phi_{I\to \mathrm{II},nm}\left(r_{\mathrm{II}};D\right)+\phi_{\mathrm{II}}\left(r_{\mathrm{II}}-S\right)$$

Based on Equation (1), the following design relates the axial spacing *D* and the lateral translation ***S*** to the beam output angle and illumination coverage of the beam generated by each MS I subaperture.

For distant or weak‐return regions, collimated beam illumination confines the projected optical power within a narrow angular range and preserves high irradiance at the target. At the design spacing *D*_0_, we require the output from each MS I subaperture to remain collimated as MS II is laterally translated. The combined output phase must therefore remain linear in the transverse coordinates, with its gradient determining the beam direction. To meet this requirement, we design MS II with the quadratic phase *ϕ*_II_(***r***_II_) = *p*|| ***r***_II_||^2^ and prescribe the complementary equivalent phase *ϕ*_I→II,_ *_nm_* (***r***_II_; *D*_0_) = −*p*|| ***r***_II_ − ***c*** *_nm_* ||^2^ for the wavefront propagated from each MS I subaperture to the MS II plane, where *p* is the constant phase coefficient. Substituting these phase profiles into Equation (1) gives

(2) $$\phi_{\mathrm{out},nm}\left(r_{\mathrm{II}};D_{0},S\right)=2p\left(c_{nm}-S\right)\cdot r_{\mathrm{II}}+p\left(∥S{∥}^{2}-∥c_{nm}{∥}^{2}\right)$$

Equation (2) shows that the quadratic terms cancel, leaving a linear phase term and a spatially constant term. The first term corresponds to a uniform transverse phase gradient, ensuring collimated output and determining the beam output angle for the beam generated by the (*n*, *m*)‐th MS I subaperture, whereas the second term is spatially constant and does not affect the beam direction. Each MS I subaperture is designed for a predefined reference direction, and the lateral translation ***S*** of MS II continuously changes the beam output angle within the corresponding FoV subregion (Figure 1b). We define the beam output angle by the output angle of the central ray passing through the center of the (*n*, *m*)‐th MS I subaperture. At the subaperture center, the wavefront propagated from MS I has zero transverse phase gradient, so the central ray reaches MS II normally and its transverse arrival position remains unchanged as the interlayer spacing varies. According to the generalized Snell's law [29], the output transverse wavevector of the central ray is therefore determined solely by the phase gradient of the laterally translated MS II and is independent of *D* (Figure 1c). The beam output angles along the two transverse directions are then given by

(3) $$\theta_{q,nm}^{c}\left(S\right)=\arcsin\left[\frac{2p\left(c_{q,nm}-S_{q}\right)}{k_{0}}\right]$$

Here and below, *k*_0_ = 2π/*λ*_0_, and *q* ∈ {*x*, *y*} denotes either transverse direction, with *c_x,nm_ = x_n_*, *c_y,nm_ = y_m_*, and *S_q_* denoting the corresponding component of ***S***.

When the interlayer spacing changes from *D*_0_ to *D*_0_ + Δ*D*, the beam output angle remains determined by Equation (3), whereas the edge rays arrive at shifted transverse positions on MS II. This axial‐spacing tuning expands the collimated beam and forms expanded beam illumination (Figure 1d). To quantify this beam expansion along the transverse direction, we consider an edge ray along the *q* direction. For collimated beam illumination at the design spacing *D*_0_, the edge ray reaches the MS II output plane with a transverse separation *r*_e_ from the central ray along the *q* direction, and the MS II phase gradient compensates the incident transverse wavevector so that the edge ray exits with the same beam output angle as the central ray. When the axial‐spacing change is Δ*D*, the transverse arrival coordinate of the edge ray on the MS II output plane changes accordingly (Figure 1e). Therefore, the axial‐spacing tuning changes the transverse arrival coordinate of the edge ray on MS II, and the magnitude of the resulting transverse wavevector change, Δ*k*(Δ*D*), is

(4) $$\Delta k\left(\Delta D\right)=\frac{4p^{2}r_{e}}{\sqrt{k_{0}^{2}-4p^{2}r_{e}^{2}}}\left|\Delta D\right|$$

Equation (4) can be written as Δ*k*(Δ*D*) = *χ*| ***Δ**D*|, where *χ* = 4*p* ^2^ *r*_e_/√(*k*_0_^2^−4*p* ^2^ *r*_e_^2^). For the beam generated by the (*n*, *m*)‐th MS I subaperture under the lateral translation ***S*** of MS II, Equation (3) corresponds to the *q*‐direction component of the transverse wavevector of the central ray, *k* ^c^ *_q_*_,_ *_nm_* = 2*p*(*c_q_*_,_ *_nm_* − *S_q_*). The two edge rays therefore have *q*‐direction components *k* ^c^ *_q_*_,_ *_nm_* ± *χ*|Δ*D*|. The output‐angle offsets between the two edge rays and the central ray define the +*q* and −*q* boundaries of the illumination coverage for the beam generated by the (*n*, *m*)‐th MS I subaperture,

(5) $$\begin{array}{ccc} & & \Omega_{q,nm}^{\pm }\left(S,\Delta D\right)\\ & & \;=\pm \left\{\arcsin\left[\frac{2p\left(c_{q,nm}-S_{q}\right)\pm \chi \left|\Delta D\right|}{k_{0}}\right]-\theta_{q,nm}^{c}\left(S\right)\right\} \end{array}$$

Here, *Ω* ^+^ *_q_*_,_ *_nm_* and *Ω* ^−^ *_q_*_,_ *_nm_* denote the output‐angle spans from *θ* ^c^ *_q_*_,_ *_nm_* to the +*q* and −*q* boundaries of the illumination coverage, respectively. The overall illumination coverage *Θ*(***S***, Δ*D*) of the TATCMs is obtained by combining the coverage regions associated with all MS I subapertures used for illumination,

(6) $$\begin{array}{c} \Theta \left(S,\Delta D\right)=\underset{n,m}{\cup }\prod_{q\in \left\{x,y\right\}}[\theta_{q,nm}^{c}\left(S\right)-\Omega_{q,nm}^{-}\left(S,\Delta D\right),\\ \theta_{q,nm}^{c}\left(S\right)+\Omega_{q,nm}^{+}\left(S,\Delta D\right)] \end{array}$$

Here, ⋃*_n_*_,_ *_m_* combines the MS I subapertures used for illumination and ∏*_q_*_∈{_ *_x_*_,_ *_y_*_}_ denotes the Cartesian product of the beam output angle intervals along the two transverse directions. Equation (6) gives a directly calculable expression for the overall illumination coverage under the lateral translation ***S*** of MS II and the axial‐spacing change Δ*D*, with the full derivation provided in Note S1.

To validate the theoretical relations established above, we design and implement TATCMs operating at 905 nm, a wavelength commonly used in active three‐dimensional sensing [30]. The target two‐dimensional FoV for collimated beam illumination is set to approximately 70°×70°. MS I is divided into 37 square MS I subapertures arranged in a truncated 7×7 array, with each subaperture associated with one local FoV subregion at the design spacing *D*_0_ (Figure 2a). The corner positions are excluded to match the approximately circular footprint of the collimated incident beam used in the experiment. MS II is designed with a lateral half‐stroke of 300 µm along both the *x* and *y* directions, corresponding to |*S_x_* | ≤ 300 µm and |*S_y_* | ≤ 300 µm. For collimated beam illumination at *D*_0_, the side length of each MS I subaperture is set to *L* = 600 µm, matching the full lateral stroke and enabling adjacent local FoV subregions to tile the target FoV without angular gaps. Under axial‐spacing tuning, the illumination coverage associated with each MS I subaperture expands, allowing neighboring coverage regions to connect with a smaller lateral stroke.

**FIGURE 2:**
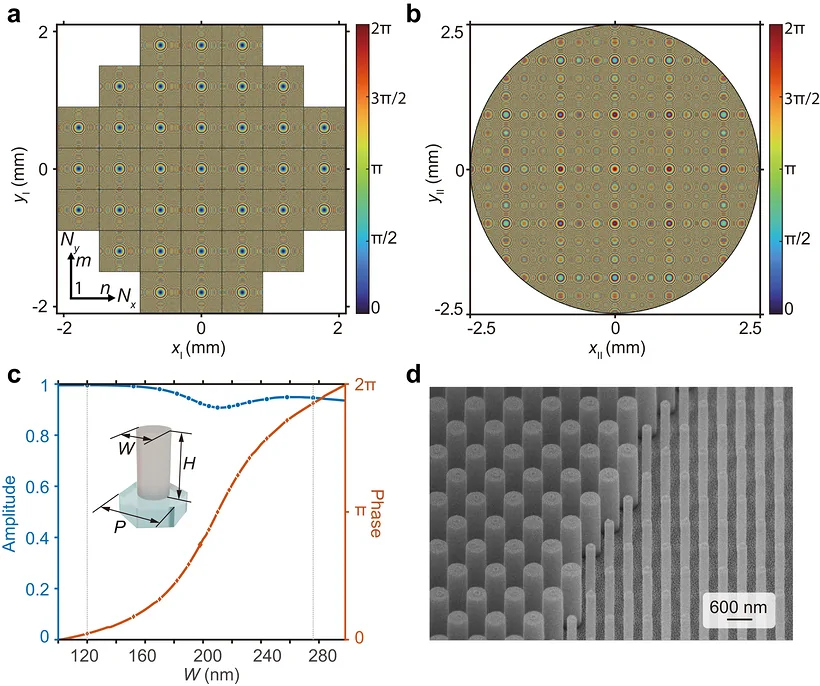
Design and implementation of TATCMs. (a) Phase profile of the first metasurface (MS I). (b) Phase profile of the second metasurface (MS II). (c) Simulated transmission amplitude and phase of amorphous‐silicon nanopillars versus width *W* at 905 nm. Circles indicate the selected meta‐atoms. (d) Scanning electron microscopy image of a local region of MS II.

The quadratic phase coefficient of MS II is determined from Equation (3) to meet the target FoV under the prescribed lateral stroke, giving *p* = −0.95 × 10^−3^ µm^−2^ (Figure 2b). The design interlayer spacing is set to *D*_0_ = 1 mm. For each MS I subaperture, we first specify the equivalent phase *ϕ*_I→II,_ *_nm_* (***r***_II_; *D*_0_) required on the MS II plane for collimated beam illumination at *D*_0_. The corresponding physical MS I phase can be constructed from this prescribed field through wave‐optics back‐propagation [31] or reverse ray tracing [23]. In this work, we adopt reverse ray tracing to construct the physical phase profile of MS I. The local gradient of *ϕ*_I→II,_ *_nm_* determines the transverse wavevector and propagation direction of each ray between MS I and MS II. We then trace the rays backward over *D*_0_ to determine their corresponding positions ***r***_I_ on MS I. The local phase gradient required for the physical MS I phase *ϕ*_I,_ *_nm_* (***r***_I_) is obtained from the tangential wavevector change at these positions according to the generalized Snell's law. Fitting and integrating the calculated phase gradients over each subaperture gives the continuous phase profile *ϕ*_I,_ *_nm_* (***r***_I_). The detailed phase‐construction procedure is provided in Note S1. The optical propagation of the designed TATCMs is further validated using wave‐optics simulations in Note S2 and Figures S1 and S2. Equation (6) gives a maximum axial‐spacing change of Δ*D* = 2.64 mm, at which neighboring coverage regions just meet at their boundaries across the designed FoV. At this axial‐spacing change, the expanded beam illumination associated with the MS I subapertures forms connected coverage across the entire 70°×70° target FoV even when MS II is not laterally translated. TATCMs are implemented using polarization‐insensitive amorphous‐silicon nanopillars on fused‐silica substrates, with the nanopillar geometries selected by full‐wave simulations to provide high transmission and full 0–2π phase coverage at 905 nm (Figure 2c). The spectral response of the TATCMs is evaluated in Note S3 and Figures S3 and S4. The nanopillars are fabricated by standard nanofabrication processes, and scanning electron microscopy confirms their morphology after pattern transfer (Figure 2d and Methods). The performance of the TATCMs under oblique incidence is further evaluated in Note S4 and Figures S5 and S6.

### 2.2 Characterization of Adaptive Active Illumination with TATCMs

We experimentally characterize adaptive active illumination with TATCMs at 905 nm by measuring the beam output angle controlled by the lateral translation of MS II and the illumination coverage controlled by axial‐spacing tuning (Methods). A collimated incident beam is transmitted through MS I and MS II, and the output illumination is recorded on a light screen using an infrared camera. We first vary the interlayer spacing *D* to characterize axial‐spacing tuning. Figure 3a shows the output illumination recorded at four axial spacings. When *D* = *D*_0_ = 1 mm, the output forms collimated beam illumination associated with the MS I subapertures, with a measured beam divergence of 0.06°. Increasing *D* broadens the illumination coverage associated with each MS I subaperture and transforms the output from collimated beam illumination into expanded beam illumination. The states with Δ*D* = 1.2 mm and Δ*D* = 1.6 mm show a gradual expansion of the illumination coverage while the beam output angles remain nearly unchanged. At Δ*D* = 2.64 mm, neighboring illumination coverage regions meet at their boundaries and form a connected illumination state across the designed FoV. The measured optical efficiency, defined as the ratio of the optical power in the designed output illumination to the optical power incident on MS I, is 64.8% (Methods).

**FIGURE 3:**
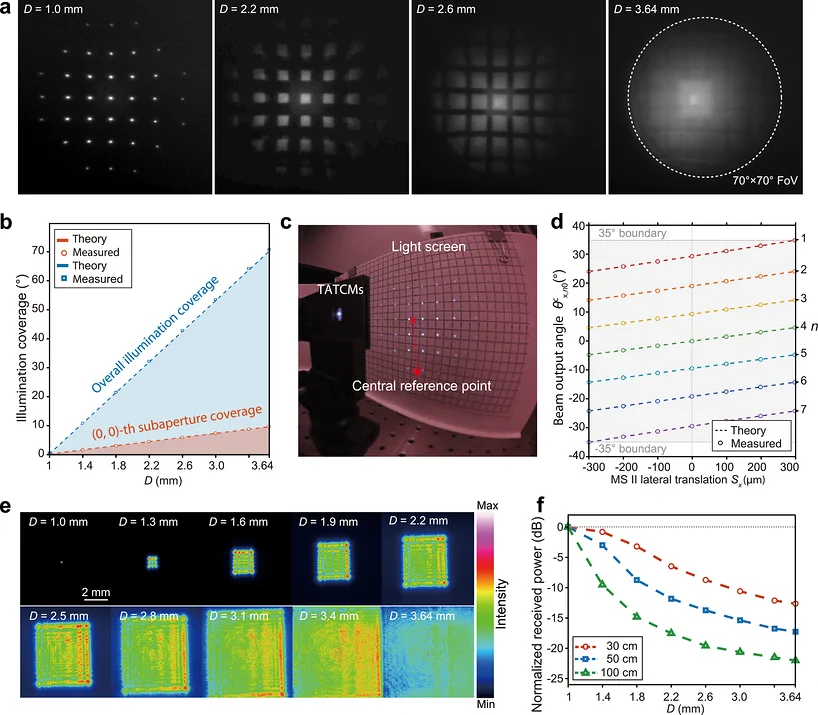
Characterization of adaptive active illumination with TATCMs. (a) Output illumination recorded on a light screen at different interlayer spacings *D*. (b) Measured and calculated illumination coverage as a function of *D*, including the coverage associated with a single MS I subaperture and the overall coverage. (c) Photograph of the setup for measuring the beam output angle on a light screen. (d) Beam output angle as a function of the MS II lateral translation for different *x*‐direction MS I subaperture indices *n*. (e) Measured beam profiles of the central illumination region at different interlayer spacings. (f) Normalized received power as a function of *D* at different propagation distances.

We then quantify the illumination coverage as the interlayer spacing *D* increases (Figure 3b). We define the central illumination region as the output illumination associated with the central MS I subaperture (*n*, *m*) = (0, 0). The illumination coverage of the central illumination region is calculated from Equation (5), and the overall illumination coverage of the TATCMs is obtained from Equation (6). The calculated results are compared with measurements extracted from the output illumination recorded on the light screen. The full output‐angle span of the central illumination region increases monotonically with *D*, reaching approximately 9.3° at Δ*D* = 2.64 mm. The overall illumination coverage also increases with *D*, reaching approximately 70° at *D* = 3.64 mm and corresponding to the full 70°×70° two‐dimensional FoV. At this spacing, neighboring illumination regions connect and provide continuous coverage of the designed FoV. Further increasing *D* broadens the illumination coverage beyond the designed FoV and increases the overlap between neighboring illumination regions, while reducing the local optical power density (Note S5 and Figure S7). The measured coverage agrees well with the calculated results, validating the illumination‐coverage relations described by Equations (5) and (6).

We next characterize beam‐output‐angle control by laterally translating MS II along the *x* direction. Equation (3) uses the central ray to represent the beam output angle, so the measurement is performed at *D*_0_, where collimated beam illumination allows direct identification of the beam centers on the light screen (Figure 3c). During the measurement, MS II is translated along *x* over the ±300 µm lateral stroke, while the *y*‐direction translation is fixed. Figure 3d shows the measured beam output angle *θ* ^c^ *_x_*_,_ *_n_*_0_ for the *m* = 0 row of MS I subapertures. As *S_x_* increases, *θ* ^c^ *_x_*_,_ *_n_*_0_ changes continuously within each local FoV subregion. The ±300 µm lateral stroke allows each MS I subaperture to sweep its assigned local FoV subregion. The swept output‐angle intervals of neighboring MS I subapertures connect without angular gaps, producing continuous coverage across the approximately 70°×70° FoV. The measured results agree well with the theoretical values calculated from Equation (3), validating the beam‐output‐angle relation controlled by the lateral translation of MS II.

We further examine the central illumination region to evaluate the local beam cross section and received‐power variation during axial‐spacing tuning. We record two‐dimensional intensity profiles of the central illumination region at a propagation distance of 15 cm as *D* increases from *D*_0_ to 3.64 mm (Figure 3e). At *D* = *D*_0_, the central illumination region appears as a collimated‐beam intensity distribution. With increasing *D*, the measured intensity distribution broadens while maintaining a relatively uniform intensity over the expanded region, corresponding to expanded beam illumination with larger illumination coverage. We then place the finite‐area probe of an optical power meter at propagation distances of 30, 50 and 100 cm to measure the optical power collected from the central illumination region (Figure 3f). When *D* exceeds approximately 1.4 mm, the expanded illumination becomes larger than the probe received area, so the measured power decreases.

The received‐power measurement shows that axial‐spacing tuning can adapt illumination coverage and local received power to sensing distance. Expanded beam illumination increases the illumination coverage obtained in each projection step and can reduce the number of scanning steps required to cover a given FoV. In contrast, collimated beam illumination concentrates optical power within a smaller illumination region and is more suitable for small or distant targets that require higher local received power. For the characterized TATCMs, the illumination coverage associated with each MS I subaperture increases nearly linearly with the axial‐spacing change Δ*D*. For a fixed local FoV subregion, the required number of angular sampling points is approximately inversely proportional to the covered angular area. The angular sampling efficiency therefore scales approximately as Δ*D* ^2^ (Note S6 and Figure S8). Beam expansion enlarges the illuminated region and thereby lowers the local optical power density for the same projected optical power. For small or distant targets, the optical power delivered to the effective target area decreases, and the maximum detectable distance scales approximately as Δ*D* ^−1/2^ for a fixed detection threshold (Note S7 and Figure S9). These two scaling relations quantify the trade‐off in adaptive active illumination, in which increasing illumination coverage improves angular sampling efficiency but reduces the maximum detectable distance for small or distant targets. A quantitative comparison with fixed expanded illumination and narrow‐beam scanning is also provided (Note S8, Table S1, and Figures S10 and S11). By combining the lateral translation of MS II for beam‐output‐angle control with axial‐spacing tuning for illumination‐coverage control, TATCMs can continuously adjust the balance between angular sampling efficiency and local power concentration according to target distance, reflectivity, size and local geometry in active three‐dimensional sensing.

### 2.3 Adaptive Three‐Dimensional Sensing Demonstration

We construct a direct time‐of‐flight (dToF) sensing system by combining the TATCMs with a commercial receiver based on a single‐photon avalanche diode (SPAD) array (Figure 4a). We collimate the output of a 905 nm vertical‐cavity surface‐emitting laser (VCSEL) and direct the beam through the TATCMs. We reconstruct the three‐dimensional point cloud from the returned signals recorded by the receiver. The system calibration and point‐cloud reconstruction procedures are described in Note S9 and Figure S12. The target scene contains two reflective white toys at near and intermediate distances and a low‐reflectivity black cube at a longer distance (Figure 4b). The differences in target distance and reflectivity require different illumination states. Expanded beam illumination supports rapid acquisition of the near and intermediate‐distance objects, whereas illumination with a smaller coverage concentrates optical power on the distant low‐reflectivity cube and strengthens the returned signal. In this experiment, the region containing the low‐reflectivity cube was predefined, and the illumination settings and rescan trajectory were tailored to adaptively enhance depth reconstruction in this region.

**FIGURE 4:**
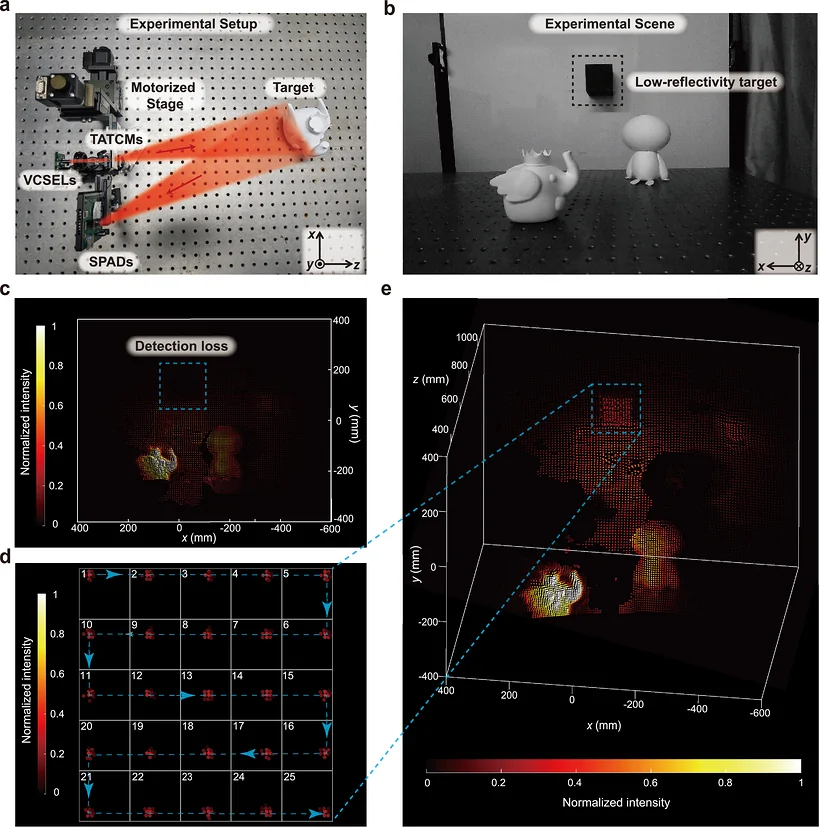
Adaptive three‐dimensional sensing demonstration with TATCMs. (a) Experimental setup of the direct time‐of‐flight (dToF) sensing system. (b) Camera image of the experimental scene containing two white toys at near and intermediate distances and a distant low‐reflectivity black cube. (c) Point cloud obtained with expanded beam illumination at *D* = 3.64 mm. (d) Adaptive serpentine scanning trajectory. (e) Adaptive three‐dimensional reconstruction obtained by combining expanded beam illumination with adaptive scanning.

We acquire an initial point cloud with expanded beam illumination at *D* = 3.64 mm (Figure 4c). The expanded beam illumination covers a large scene region in a single projection. The resulting reconstruction contains the near and intermediate‐distance toys. The distant low‐reflectivity cube is absent from the reconstruction because the large illumination area reduces the optical power incident on the cube and weakens the returned signal. The absence of the cube from the point cloud indicates that the corresponding scene region requires higher local power concentration.

Then, we reduce the interlayer spacing *D* from 3.64 to 1.60 mm to decrease the illumination coverage and increase the optical power delivered to the cube. At *D* = 1.60 mm, the reduced illumination coverage produces a distinct returned signal from the distant low‐reflectivity cube. Based on the measured illumination coverage and the relation between illumination coverage and the required number of angular samples, we select 25 scanning positions to cover the target region. The scanning positions follow a programmed serpentine trajectory, and the acquired measurements reconstruct the distant cube (Figure 4d). The resulting adaptive three‐dimensional reconstruction contains the toys at near and intermediate distances and the distant low‐reflectivity cube (Figure 4e). We compare the reconstructed depths of the four visible cube vertices with reference measurements, obtaining a mean relative depth error of 2.17%, and further assess the spatial variation of depth deviations across the reconstructed scene (Note S9 and Figure S13). The reconstruction results verify that axial‐spacing tuning adjusts illumination coverage and local power concentration according to target distance and reflectivity. The present demonstration establishes the potential of TATCMs for adaptive illumination. Fully autonomous scene‐dependent adaptation would require measurement feedback, decision‐making, and closed‐loop illumination control, as further discussed in Note S10 and Figure S14.

## 3 Conclusions

In this work, we have demonstrated adaptive active illumination with TATCMs by combining beam‐output‐angle control through lateral translation with illumination‐coverage control through axial‐spacing tuning. By controlling both the beam output angle and illumination coverage, TATCMs adjust the illumination coverage obtained in each projection and enable a tunable trade‐off between maximum detectable distance and angular sampling efficiency. We have implemented the TATCMs at 905 nm and experimentally characterized their adaptive active‐illumination performance. Lateral translation provides continuous beam‐output‐angle control across an approximately 70°×70° FoV under a ±300 µm lateral stroke. Axial‐spacing tuning continuously expands the illumination coverage, from collimated beam illumination with a measured divergence of 0.06° to continuous coverage across the designed FoV after a 2.64 mm increase in interlayer spacing. We have further demonstrated adaptive three‐dimensional reconstruction by selecting the illumination state according to sensing requirements, verifying the practical relevance of this distance‐efficiency trade‐off. These results show that TATCMs provide a compact planar route for adaptive active illumination in three‐dimensional sensing, where illumination coverage and local power concentration can be adjusted according to scene‐specific sensing requirements. A comparison with representative reconfigurable beam‐steering and mechanically actuated approaches is provided in Note S11 and Tables S2 and S3. This capability could benefit autonomous vehicles, unmanned aerial vehicles and industrial robots operating across targets with varying distances and reflectivities.

## 4 Methods

### 4.1 Simulation and Fabrication of TATCMs

Full‐wave electromagnetic simulations were performed to calculate the nanopillar response at 905 nm. The simulated structure was a polarization‐insensitive amorphous‐silicon nanopillar on a fused‐silica substrate. The nanopillar height and hexagonal lattice period were fixed at 600 and 450 nm, respectively, and the nanopillar width *W* was varied from 120 to 276 nm to obtain the transmitted phase and amplitude. Periodic boundary conditions were applied in the lateral directions, and open boundaries were used along the propagation direction. The selected nanopillar geometries provided full 0–2π phase coverage with simulated transmission amplitude above 90%, and were used to discretize the designed MS I and MS II phase profiles into fabrication layouts. For fabrication, a 600‐nm‐thick amorphous‐silicon film was deposited on fused‐silica substrates by plasma‐enhanced chemical vapor deposition (PECVD). The metasurface patterns were defined by electron‐beam lithography (EBL) and transferred into the amorphous‐silicon layer by inductively coupled plasma etching.

### 4.2 Characterization of Adaptive Active Illumination with TATCMs

The optical characterization was performed using a 905 nm fiber‐coupled laser source (WSLS‐905‐050m‐4, WaveSpectrum Laser). The laser beam was collimated by an adjustable fiber collimator (RADFCB‐7.5‐APC, LBTEK Optics) and directed onto the TATCMs. The output illumination was projected onto a far‐field screen and recorded by an infrared camera to characterize illumination‐coverage tuning under different axial‐spacing changes and beam‐output‐angle control under different lateral translations of MS II. Local beam profiles were measured at propagation distances of 15 and 30 cm using a beam profiler (BP209IR1/M, Thorlabs), from which the beam center and full width at half maximum were extracted to calculate the beam divergence.

### 4.3 Optical‐Efficiency Measurement of TATCMs

The optical efficiency was measured in the collimated‐illumination state at the design interlayer spacing *D* = 1 mm using an optical power meter (GCI‐08, Daheng Optics). The optical efficiency was defined as *η* = *P*_out_/*P*_in_, where *P*_in_ is the total optical power incident on MS I and *P*_out_ is the total optical power contained in the designed output spots. The residual zero‐order and other undesired background light were excluded from *P*_out_. The incident optical power was first measured before MS I, yielding *P*_in_ = 5.3 mW. The TATCMs were then operated at *D* = 1 mm, and the optical power of each designed output spot was measured individually at a plane approximately 15 cm downstream from the device. The measured powers were summed to obtain *P*_out_ = 3.435 mW, corresponding to an optical efficiency of 64.8%.

### 4.4 DToF Sensing System and Adaptive Point‐Cloud Reconstruction

A commercial SPAD‐based dToF module (VI4331, VisionICs) was adopted to construct the active three‐dimensional sensing system. The output of the integrated 905 nm VCSEL transmitter was collimated by a lens module (RADFCB‐7.5‐PC, LBTEK Optics) and directed through the TATCM illumination module. The TATCMs were mounted on motorized translation stages that independently controlled the lateral displacement of MS II and the axial spacing between MS I and MS II. Before three‐dimensional reconstruction, the system was calibrated to correct the range offset and transmitter–receiver misalignment. Depth measurements acquired under different illumination states were then transformed into point clouds in a common calibrated coordinate system. The locally scanned point cloud was subsequently fused with the expanded‐beam survey point cloud to obtain the adaptive three‐dimensional reconstruction.

## Author Contributions

C.Z. conceived the project and develops the theoretical framework. C.Z. and J.Y. performed the optical design, experiments and data analysis. L.Z. contributed to the theoretical analysis and interpretation of the results. J.B., B.L. and Q.F. contributed to the experimental measurements and data processing. R.Y. and X.J. contributed to device fabrication and structural characterization. C.Z. prepared the original manuscript. L.Z., F.X., X.Z. and Z.Y. supervised the project and revised the manuscript. All authors discussed the results and approved the final manuscript.

## Conflicts of Interest

The authors declare no conflicts of interest.

The data that support the findings of this study are available from the corresponding author upon reasonable request.

## Supporting information




**Supporting File**: advs78448‐sup‐0001‐SuppMat.pdf.
