# Supplementary material for: Adaptive Active Illumination Via Translationally and Axially Tunable Cascaded Metasurfaces
Source: Adv Sci (Weinh). 2026 Oct 27:e78448. Online ahead of print. doi: 10.1002/advs.78448 (PMC13712390; doi:10.1002/advs.78448)
Supplement: Supplementary file 1 [file ADVS-9999-e78448-s001.pdf]

Supplementary Information for

Adaptive active illumination via translationally and axially tunable cascaded metasurfaces

Chi Zhang,[1, 2, †] Jianing Yang,[1, 2, †] Lingyun Zhang,[3, *] Jingyu Bao, [4] Bingbai Li,[1, 2] Qingsong Feng,[1, 2] Rui You,[5] Xiaoli Jing,[5] Fei Xing,[1, 2, 6, *] Xiaoguang Zhao[1, 2, 6, *] and Zheng You[3, *]

[1] State Key Laboratory of Precision Measurement Technology and Instrument, Tsinghua University, Beijing, 100084, China.

[2] Department of Precision Instrument, Tsinghua University, Beijing, 100084, China.

[3] State Key Laboratory of Intelligent Manufacturing Equipment and Technology, School of Mechanical Science and Engineering, Huazhong University of Science and Technology, Wuhan, 430074, China.

[4] Beijing Institute of Tracking and Telecommunications Technology, Beijing, 100194, China.

[5] Laboratory of Intelligent Microsystems, Beijing Information Science and Technology University, Beijing, 100192, China.

[6] Beijing Advanced Innovation Center for Integrated Circuits, Tsinghua University, Beijing, 100084, China.

[†] These authors contributed equally to this work.

*Correspondence: zhangly_imet@hust.edu.cn (L. Z.); xingfei@mail.tsinghua.edu.cn (F. X.); zhaoxg@mail.tsinghua.edu.cn (X. Z.); youz@hust.edu.cn (Z. Y.)

Supplementary Note 1: Theoretical derivation for translationally and axially tunable cascaded metasurfaces for adaptive active illumination

This note derives the theoretical relation between mechanical tuning and adaptive active illumination in translationally and axially tunable cascaded metasurfaces (TATCMs), which comprise the first metasurface (MS I) and the second metasurface (MS II). The derivation establishes the relation between the lateral translation of MS II and the beam output angle, and the relation between axial-spacing tuning and illumination coverage.

We define a common Cartesian coordinate system for MS I and MS II, with the positive z direction pointing from MS I to MS II. The MS II plane at zero lateral translation is defined as the fixed x-y reference plane. At an interlayer spacing D, the centers of MS II and MS I are located at (0, 0, 0) and (0, 0, -D), respectively. A transverse position on the MS I plane is denoted by $\boldsymbol{r}_\mathrm{I} = (x_\mathrm{I}, y_\mathrm{I})$, and a transverse position on the MS II reference plane is denoted by $\boldsymbol{r}_\mathrm{II} = (x_\mathrm{II}, y_\mathrm{II})$. MS I contains $N_x \times N_y$ square subapertures. The center of the (n, m)-th MS I subaperture is denoted by $\boldsymbol{c}_{nm} = (x_n, y_m)$, where n and m index the x and y directions, respectively. The lateral translation of MS II relative to MS I is denoted by $\boldsymbol{S} = (S_x, S_y)$.

At the design spacing D_0, collimated output requires a spatially uniform transverse phase gradient across the beam. The output phase must therefore be linear in the transverse coordinates, apart from a spatially constant term. To steer the beam while maintaining collimation, we require lateral translation to change the value of the phase gradient without introducing spatial variation in the gradient. We meet both requirements by choosing complementary quadratic profiles for the MS II phase and the equivalent phase propagated from MS I to the MS II plane. We write the MS II phase as

$$\phi_\mathrm{II}(\boldsymbol{r}_\mathrm{II}) = p \parallel \boldsymbol{r}_\mathrm{II} \parallel^2 \tag{S.1}$$

where p is the quadratic phase coefficient. When MS II is laterally translated by $\boldsymbol{S}$, the MS II phase on the fixed reference plane is expressed as $\phi_\mathrm{II}(\boldsymbol{r}_\mathrm{II} - \boldsymbol{S})$. The cascaded output phase associated with the (n, m)-th MS I subaperture is therefore

$$\phi_{\mathrm{out},nm}(\boldsymbol{r}_\mathrm{II}; D, \boldsymbol{S}) = \phi_{\mathrm{I}\to\mathrm{II},nm}(\boldsymbol{r}_\mathrm{II}; D) + \phi_\mathrm{II}(\boldsymbol{r}_\mathrm{II} - \boldsymbol{S}) \tag{S.2}$$

Equation (S.2) includes the axial spacing D through the propagated MS I wavefront and the lateral translation $\boldsymbol{S}$ through the shifted MS II phase profile. At the design spacing D_0, we prescribe the equivalent phase $\phi_{\mathrm{I}\to\mathrm{II},nm}(\boldsymbol{r}_\mathrm{II}; D_0)$ on the MS II plane for the wavefront propagated from the (n, m)-th MS I subaperture as

$$\phi_{\mathrm{I}\to\mathrm{II},nm}(\boldsymbol{r}_\mathrm{II}; D_0) = -p \parallel \boldsymbol{r}_\mathrm{II} - \boldsymbol{c}_{nm} \parallel^2 \tag{S.3}$$

The physical phase $\phi_{\mathrm{I},nm}(\boldsymbol{r}_\mathrm{I})$ of the corresponding MS I subaperture is determined from $\phi_{\mathrm{I}\to\mathrm{II},nm}(\boldsymbol{r}_\mathrm{II}; D_0)$ by reverse ray tracing over D_0. The local phase gradient of $\phi_{\mathrm{I}\to\mathrm{II},nm}$ determines the transverse wavevector of each ray between MS I and MS II. For the quadratic equivalent phase in Equation (S.3), the transverse wavevector is

$$\boldsymbol{k}_{\parallel,nm}(\boldsymbol{r}_\mathrm{II}) = -2p(\boldsymbol{r}_\mathrm{II} - \boldsymbol{c}_{nm}) \tag{S.4}$$

The transverse wavevector in Equation (S.4), together with the corresponding longitudinal wavevector, determines the propagation direction of each ray between MS I and MS II. Tracing each ray backward from the position $\boldsymbol{r}_{\mathrm{II}}$ on MS II over the design spacing D_0 gives the corresponding position $\boldsymbol{r}_{\mathrm{I}}$ on MS I

$$\boldsymbol{r}_{\mathrm{I}} = \boldsymbol{r}_{\mathrm{II}} + D_0 \frac{2p(\boldsymbol{r}_{\mathrm{II}} - \boldsymbol{c}_{nm})}{\sqrt{k_0^2 - 4p^2\|\boldsymbol{r}_{\mathrm{II}} - \boldsymbol{c}_{nm}\|^2}} \tag{S.5}$$

The propagation direction obtained from reverse ray tracing determines the local phase gradient required on MS I. According to the generalized Snell's law, the phase gradient of the (n, m)-th MS I subaperture is

$$\nabla_{\boldsymbol{r}_{\mathrm{I}}} \phi_{\mathrm{I},nm}(\boldsymbol{r}_{\mathrm{I}}) = k_{\parallel,nm}^{\mathrm{out}}(\boldsymbol{r}_{\mathrm{I}}) - k_{\parallel}^{\mathrm{in}}(\boldsymbol{r}_{\mathrm{I}}) \tag{S.6}$$

The incident beam is collimated and normal to MS I, giving $k_{\parallel}^{\mathrm{in}} = 0$. Repeating the reverse ray tracing for the sampled positions on the MS II plane provides the required phase gradients at the corresponding positions $\boldsymbol{r}_{\mathrm{I}}$ on MS I. We fit the calculated phase gradients over each subaperture and integrate the fitted distribution to obtain the continuous physical phase $\phi_{\mathrm{I},nm}(\boldsymbol{r}_{\mathrm{I}})$.

Substituting Equation (S.3) and $\phi_{\mathrm{II}}(\boldsymbol{r}_{\mathrm{II}} - \boldsymbol{S}) = p\|\boldsymbol{r}_{\mathrm{II}} - \boldsymbol{S}\|^2$ into Equation (S.2) gives

$$\begin{aligned}\phi_{\mathrm{out},nm}(\boldsymbol{r}_{\mathrm{II}}; D_0, \boldsymbol{S}) &= -p \parallel \boldsymbol{r}_{\mathrm{II}} - \boldsymbol{c}_{nm} \parallel^2 + p \parallel \boldsymbol{r}_{\mathrm{II}} - \boldsymbol{S} \parallel^2 \\ &= 2p(\boldsymbol{c}_{nm} - \boldsymbol{S}) \cdot \boldsymbol{r}_{\mathrm{II}} + p(\parallel \boldsymbol{S} \parallel^2 - \parallel \boldsymbol{c}_{nm} \parallel^2)\end{aligned} \tag{S.7}$$

Equation (S.7) shows that the quadratic terms of the propagated MS I phase and the translated MS II phase cancel, leaving a linear phase term and a spatially constant term. The cancellation holds during lateral translation because translating a quadratic phase profile changes its linear and constant terms but preserves its quadratic coefficients. The first term in Equation (S.7) corresponds to a uniform transverse phase gradient and determines the beam output angle for the (n, m)-th MS I subaperture. The uniform gradient gives the same local propagation direction across the output beam, producing collimated illumination. The second term changes only the global phase and does not affect the beam output angle. At the design spacing D_0, we therefore continuously tune the beam output angle by translating MS II while maintaining collimation. Along a selected transverse direction $q \in \{x, y\}$, the q-direction component of the transverse wave vector of the central ray is

$$k_{q,nm}^{c} = \frac{\partial \phi_{\mathrm{out},nm}}{\partial q_{\mathrm{II}}} = 2p\left(c_{q,nm} - S_q\right) \tag{S.8}$$

where $c_{q,nm}$ and S_q are the q-direction components of $\boldsymbol{c}_{nm}$ and $\boldsymbol{S}$, respectively. Applying the generalized Snell's law gives the beam output angle defined by the central ray as

$$\theta_{q,nm}^{c}(\boldsymbol{S}) = \arcsin\left[\frac{2p\left(c_{q,nm} - S_q\right)}{k_0}\right] \tag{S.9}$$

where $k_0 = 2\pi/\lambda_0$. In this work, MS II is designed with a converging quadratic phase, so the quadratic phase coefficient satisfies $p < 0$ under the coordinate and phase convention used here. As a result, increasing S_q increases the beam output angle $\theta^{c}_{q,nm}$ within the

valid steering range. For the central MS I subaperture with $c_{q,nm} = 0$, a positive lateral translation $S_q > 0$ gives $\theta^c_{q,nm} > 0$, meaning that the lateral translation of MS II toward $+q$ steers the beam toward $+q$.

When the interlayer spacing is changed from D_0 to $D_0 + \Delta D$, the central ray associated with a given MS I subaperture remains the reference ray for the beam output angle. Before MS II modulation, the central ray reaches MS II normally, with zero transverse wave-vector component. The axial-spacing change modifies the optical path length of the central ray between MS I and MS II, but does not change its transverse arrival coordinate on MS II. The beam output angle therefore remains described by Equation (S.9). The edge rays at the opposite sides of the same MS I subaperture have nonzero transverse wave-vector components before reaching MS II. Changing the interlayer spacing shifts their transverse arrival coordinates on MS II. Because the MS II phase profile is fixed, these shifted arrival coordinates correspond to different local phase gradients and produce output-angle offsets from the central ray. These offsets define the illumination coverage generated by axial-spacing tuning.

To quantify this beam expansion along a principal transverse direction q, we consider the two edge rays on the opposite sides of the central ray. For collimated beam illumination at the design spacing D_0, the central ray and an edge ray arrive at the MS II output plane with a transverse separation r_e along the q direction. At this spacing, the MS II phase gradient compensates for the incident transverse wave-vector difference between the central ray and the edge ray, so the edge ray exits with the same beam output angle as the central ray. At the design spacing D_0, the transverse wave-vector component of this edge ray before reaching MS II has the magnitude $2|p|r_e$. The corresponding longitudinal wave-vector component is therefore

$$k_z(r_e) = \sqrt{k_0^2 - 4p^2 r_e^2} \tag{S.10}$$

When the interlayer spacing changes by ΔD, the edge ray propagates over an additional axial distance with the same local wave-vector direction in the interlayer region. The transverse separation between the edge ray and the central ray on the MS II plane therefore changes by

$$|\Delta r_e| = \frac{2|p|r_e}{\sqrt{k_0^2 - 4p^2 r_e^2}} |\Delta D| \tag{S.11}$$

The changed transverse separation makes the edge ray sample a different position on the fixed MS II phase profile, and the magnitude of the resulting transverse wave-vector change is

$$\Delta k(\Delta D) = 2|p||\Delta r_e| = \frac{4p^2 r_e}{\sqrt{k_0^2 - 4p^2 r_e^2}} |\Delta D| \tag{S.12}$$

Equation (S.12) gives the magnitude of the transverse wave-vector change for an edge ray. The two edge rays on opposite sides of the central ray give opposite signs of this change. For compact notation, we write $\Delta k(\Delta D) = \chi|\Delta D|$, where $\chi = 4p^2 r_e/\sqrt{(k_0^2 - 4p^2 r_e^2)}$. For the beam associated with the (n, m)-th MS I subaperture, the q-direction transverse wave-vector components of the two edge rays after MS II are therefore

$$k_{q,nm}^{\pm} = k_{q,nm}^{c} \pm \chi|\Delta D| = 2p\left(c_{q,nm} - S_q\right) \pm \chi|\Delta D| \tag{S.13}$$

The output angle of each edge ray is determined by its q-direction transverse wave-vector component after MS II. The positive output-angle offsets from the central ray to the two illumination boundaries are therefore

$$\Omega_{q,nm}^{\pm}(\boldsymbol{S}, \Delta D) = \pm\left\{\arcsin\left[\frac{2p\left(c_{q,nm} - S_q\right) \pm \chi|\Delta D|}{k_0}\right] - \theta_{q,nm}^{c}(\boldsymbol{S})\right\} \tag{S.14}$$

Here, $\Omega^{+}_{q,nm}$ denotes the offset from the central ray to the $+q$ edge, and $\Omega^{-}_{q,nm}$ denotes the offset from the central ray to the $-q$ edge. The outer sign in Equation (S.14) makes both quantities positive. The inner sign corresponds to the two edge rays on the opposite sides of the central ray.

For the (n, m)-th MS I subaperture, the illumination coverage is represented by the output-angle interval bounded by the two edge rays along each transverse direction. In two-dimensional operation, this coverage is the Cartesian product of the intervals along x and y. Combining the coverage regions associated with all MS I subapertures used for illumination gives the overall illumination coverage of the TATCMs,

$$\Theta(\boldsymbol{S}, \Delta D) = \bigcup_{n,m} \prod_{q\in\{x,y\}} \left[\theta_{q,nm}^{c}(\boldsymbol{S}) - \Omega_{q,nm}^{-}(\boldsymbol{S}, \Delta D),\; \theta_{q,nm}^{c}(\boldsymbol{S}) + \Omega_{q,nm}^{+}(\boldsymbol{S}, \Delta D)\right] \tag{S.15}$$

where $\bigcup_{n,m}$ combines the MS I subapertures used for illumination, and $\prod_{q\in\{x,y\}}$ denotes the Cartesian product of the beam output angle intervals along the two transverse directions. Equation (S.15) gives the overall illumination coverage under the lateral translation $\boldsymbol{S}$ of MS II and the axial-spacing change ΔD.

Supplementary Note 2: Wave-optics validation of TATCMs

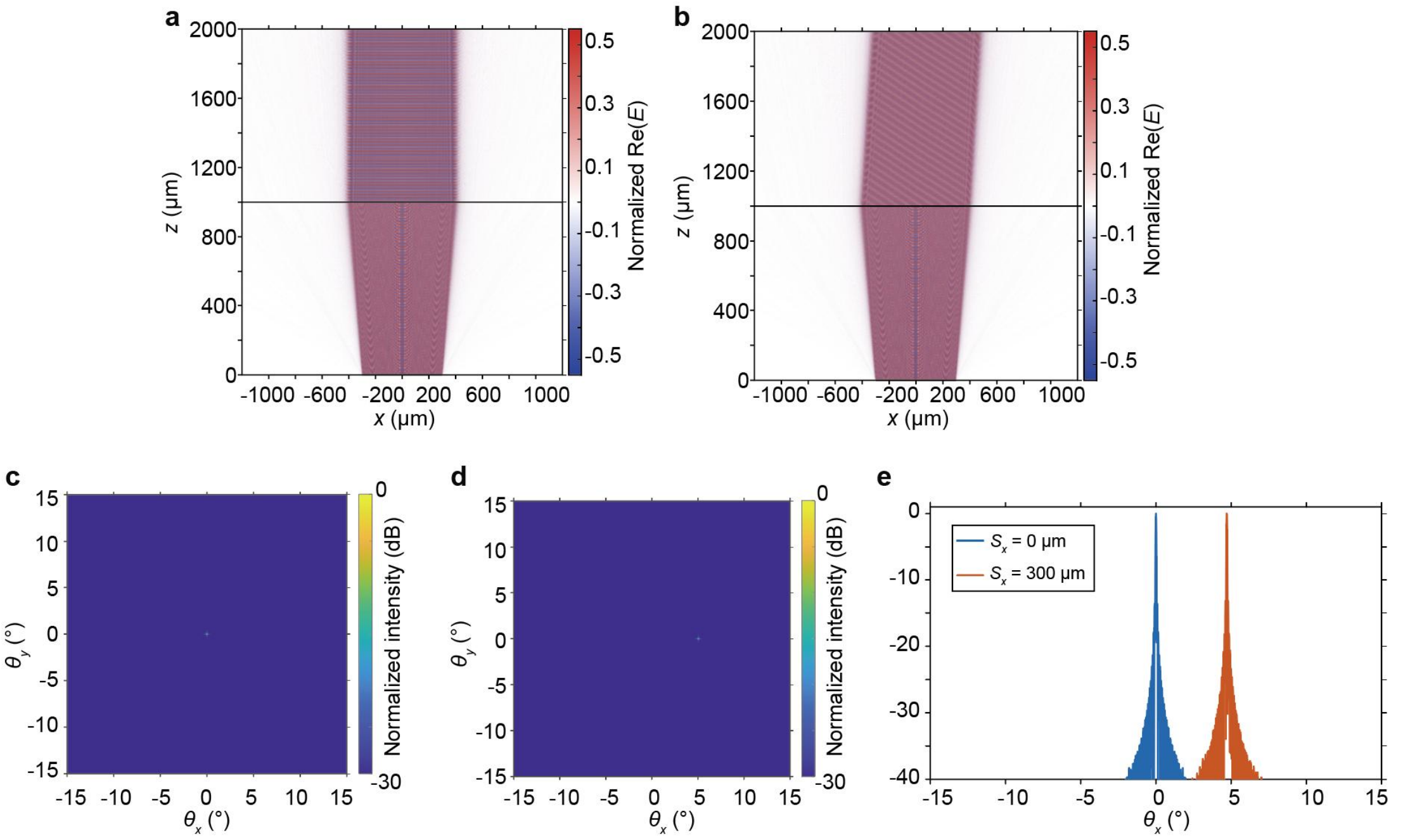

Supplementary Figure S1 | Wave-optics simulation of lateral beam steering in the TATCMs. **a,b**, Simulated *x*–*z* field distributions at D = 1 mm for S_x = 0 and S_x = 300 μm, respectively. **c,d**, Corresponding two-dimensional far-field angular intensity distributions. **e,** Normalized far-field angular intensity profiles along θ_x.

To further validate the optical propagation of the TATCMs beyond the unit-cell simulations, we performed a multiscale wave-optics simulation that connects the discrete metasurface phase distributions with free-space propagation between MS I and MS II. Direct three-dimensional full-wave simulation of the entire device is computationally prohibitive because the metasurfaces extend over the millimeter scale while the constituent unit cells have a subwavelength period of 450 nm. We therefore retain the actual device dimensions and describe each unit cell through its local complex transmission coefficient $t_j = A_j \exp(i\phi_j)$.

We consider one complete MS I subaperture with a lateral size of 600 μm and the corresponding region of MS II. The actual unit-cell period of 450 nm is retained. The incident field immediately after MS I is expressed as $U_{\mathrm{I}}(x,y) = U_0(x,y)t_{\mathrm{I}}(x,y)$. The field is then propagated over the interlayer spacing D using the angular-spectrum method. In the spatial-frequency domain, the propagated field is given by $\widetilde{U}(k_x,k_y,D) = \widetilde{U}(k_x,k_y,0)\exp(ik_zD)$, where $k_z = \sqrt{k_0^2 - k_x^2 - k_y^2}$. The propagated field is subsequently modulated by MS II according to its laterally translated phase distribution and is further propagated to obtain the output field. The far-field angular distribution is calculated from the spatial Fourier spectrum of the complex field after MS II.

Supplementary Figure S1 shows representative results for lateral translation at the

design spacing $D = 1$ mm. For $S_x = 0$, the output wavefront after MS II is approximately planar and the far-field distribution is centered near the optical axis. When MS II is translated to $S_x = 300$ μm, the output wavefront becomes tilted and the far-field distribution shifts accordingly. The simulated steering angle is approximately 4.70°, which agrees closely with the theoretical value of 4.71°. The wave-optics simulation therefore reproduces the lateral-translation-controlled beam-steering relation derived in the main text.

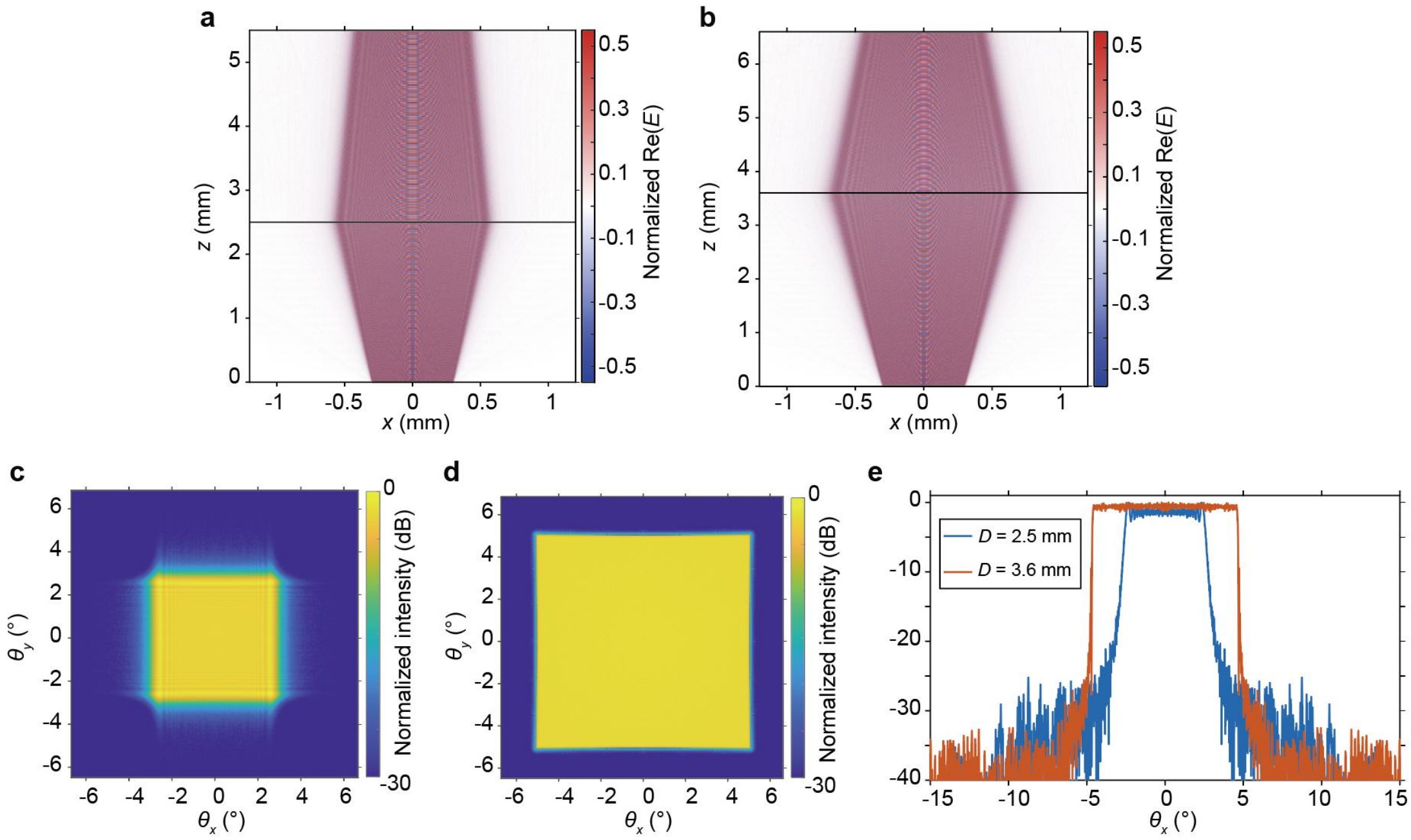

Supplementary Figure S2 | Wave-optics simulation of axial illumination tuning in the TATCMs. a,b, Simulated *x*–*z* field distributions at $S_x = 0$ for $D = 2.5$ mm and $D = 3.6$ mm, respectively. **c,d**, Corresponding two-dimensional far-field angular intensity distributions. **e,** Normalized far-field angular intensity profiles along θ_x.

Supplementary Figure S2 shows the corresponding results for axial-spacing tuning at $S_x = 0$. When the interlayer spacing is increased from $D = 2.5$ mm to $D = 3.6$ mm, the wavefront incident on MS II changes because of the additional free-space propagation between the two metasurfaces. After modulation by MS II, the output field first converges and subsequently diverges during further propagation. The associated far-field angular distribution broadens as *D* increases, corresponding to an increased illumination coverage. This behavior agrees with the theoretical relation between axial spacing and illumination coverage established in the main text.

The simulated field distributions also show that diffraction-induced distortions remain relatively weak under the present device parameters. The 600 μm subaperture is much larger than the operating wavelength, while the 450 nm lattice period remains subwavelength at 905 nm. The propagation is therefore dominated by the designed propagating-wave components. The agreement between the wave-optics simulations and the theoretical predictions provides an independent device-scale validation of both lateral-translation-controlled beam steering and axial-spacing-controlled illumination coverage.

Supplementary Note 3: Spectral response of TATCMs

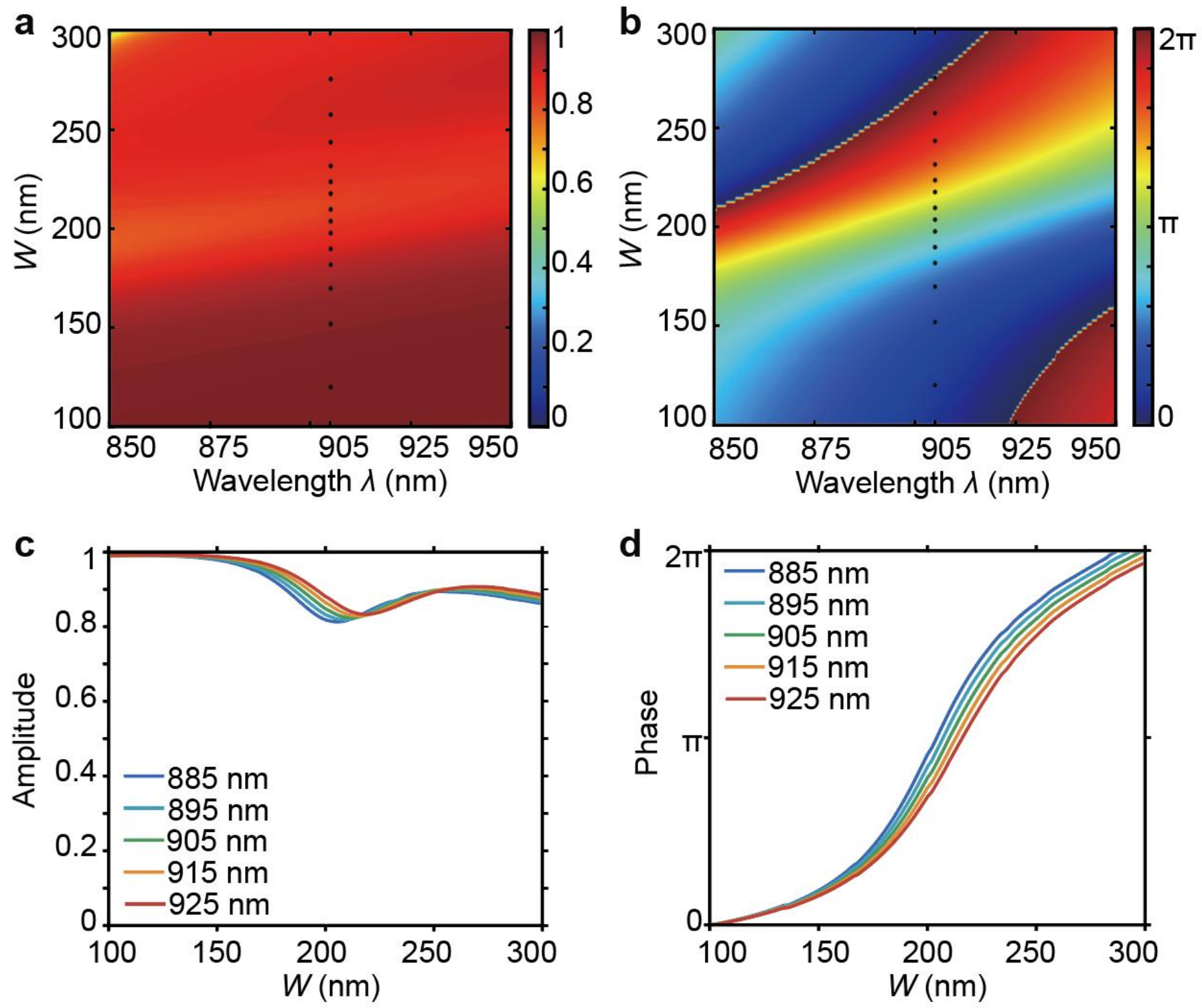

Supplementary Figure S3 | Spectral response of the selected meta-atom library. **a,b,** Simulated transmission amplitude and phase as functions of wavelength and nanopillar width W. **c,d,** Transmission amplitude and phase as functions of W.

The TATCMs were designed and experimentally implemented at 905 nm. To evaluate the influence of finite source bandwidth, we considered a wavelength range of 885–925 nm, corresponding to ±20 nm around the design wavelength. This range is substantially broader than the typical emission linewidth of practical 905-nm VCSELs and pulsed semiconductor lasers and therefore provides a conservative spectral-stability assessment. Figure S3 presents the spectral response of the selected meta-atom library. Figure S3a,b show the complete wavelength-dependent transmission amplitude and phase obtained from the same full-wave unit-cell simulations used for Figure 2c in the main text. The black markers indicate the meta-atoms selected for the 905-nm design. Figure S3c,d further show the transmission amplitude and phase as functions of nanopillar width at 885, 895, 905, 915, and 925 nm. The responses evolve smoothly over this wavelength range.

To evaluate the wavelength dependence of the beam output angle, the phase responses at different wavelengths were described by $\phi_i(\lambda) \approx a(\lambda)\phi_i(905) + b(\lambda)$, where $a(\lambda)$ represents the scaling of the effective phase gradient and $b(\lambda)$ is a common phase offset. The central MS I subaperture $(n, m) = (0, 0)$ was selected as a representative channel. The wavelength-dependent phase-gradient scaling was introduced into the beam-output-angle relation in Equation (3) of the main text. As shown in Figure S4a, the maximum wavelength-induced deviation from the 905-nm

response over the full S_x = ±300 μm range is 0.133°, corresponding to approximately 2.8% of the 905-nm steering angle at the edge of the scan range.

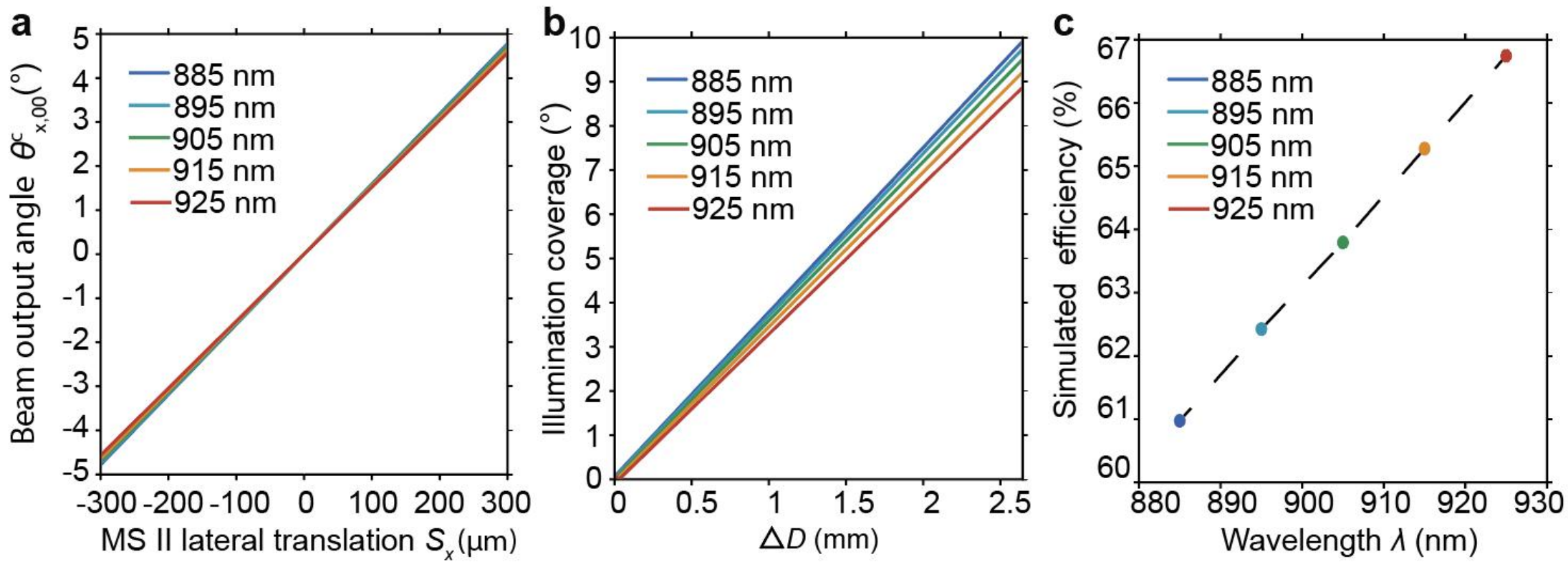

Supplementary Figure S4 | Wavelength dependence of TATCM performance. a, Beam output angle as a function of MS II lateral translation. **b,** Illumination coverage as a function of axial-spacing change. **c,** Simulated transmission efficiency as a function of wavelength.

Using the same central MS I subaperture, we further evaluated the wavelength dependence of the illumination coverage. For each wavelength, the effective quadratic phase coefficient was scaled according to the corresponding phase response, and the wavelength-dependent parameters were substituted into Equations (4) and (5) of the main text. As shown in Figure S4b, at ΔD = 2.64 mm, the maximum relative deviation from the 905-nm illumination coverage among the five wavelengths is 6.71%.

The wavelength dependence of the transmission was also evaluated using the same full-wave unit-cell simulation results. For each wavelength, the power transmittances of the selected meta-atoms were obtained from their simulated transmission amplitudes, and the average transmission was used to estimate the two-layer transmission of the cascaded metasurfaces. As shown in Figure S4c, the estimated transmission efficiency varies from 60.97% to 66.74% over 885–925 nm, with a maximum relative deviation of 4.63% from the value at 905 nm.

Overall, the wavelength-induced variations in beam output angle, illumination coverage, and transmission remain moderate over a spectral range substantially broader than the typical linewidth of practical 905-nm sources. The corresponding variations are therefore expected to be smaller under practical narrowband 905-nm operation.

Supplementary Note 4: Oblique-incidence performance of TATCMs

The TATCMs are designed under normal incidence. We further evaluate their performance under external oblique incidence because a finite incidence angle introduces an additional transverse wave-vector component before MS I and consequently changes the propagation trajectory between MS I and MS II.

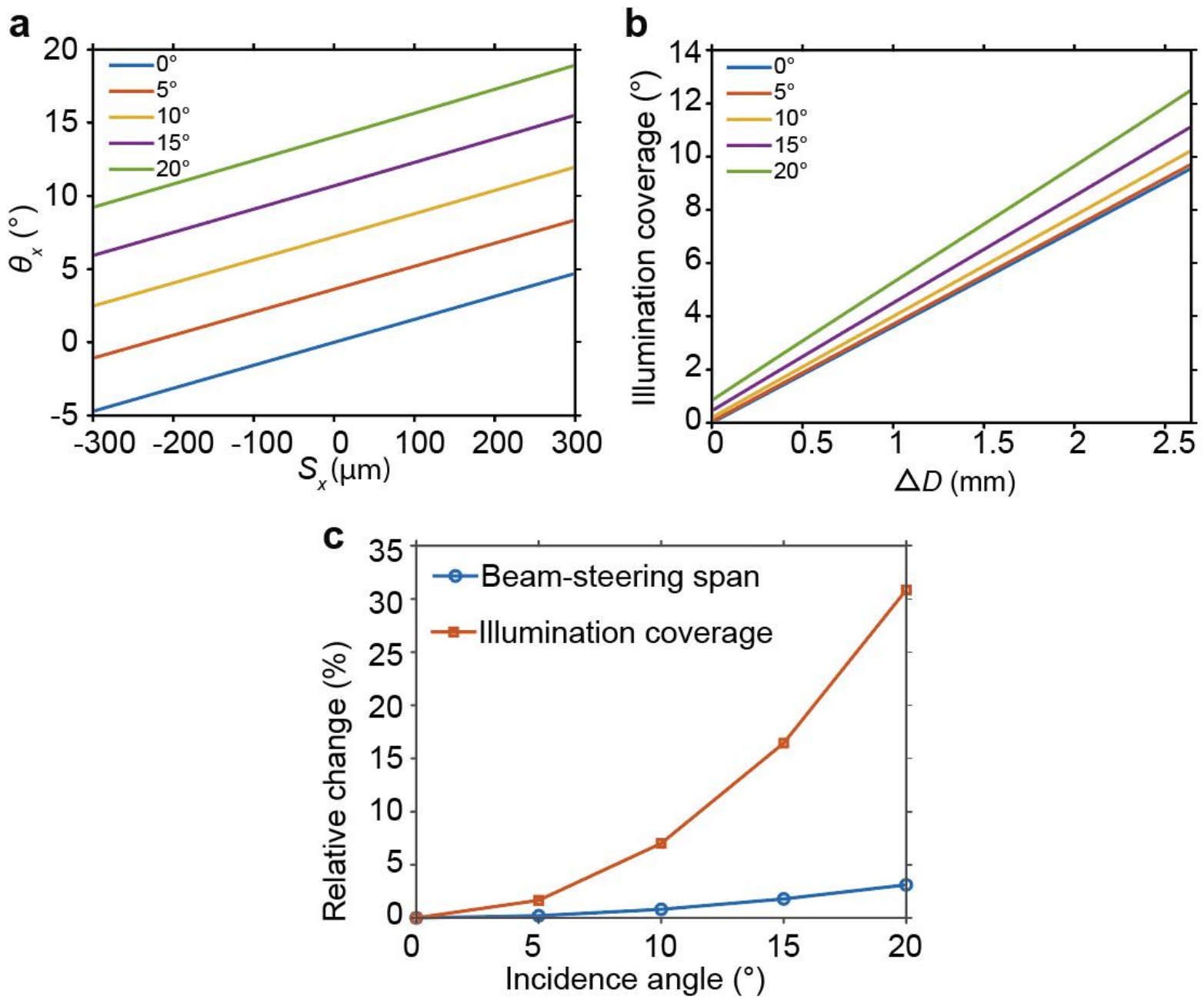

Supplementary Figure S5 | Oblique-incidence sensitivity of the TATCMs. a, Beam output angle θ_x as a function of lateral translation S_x under external incidence angles from 0° to 20°. **b**, Illumination coverage as a function of axial-spacing variation ΔD under the same incidence angles. **c**, Relative changes in the beam-steering span and illumination coverage as functions of the external incidence angle, referenced to normal incidence.

We first examine the angular response of the meta-atoms and the system-level tuning characteristics. Full-wave simulations show that the selected a-Si meta-atoms maintain an overall stable phase response as the incidence angle increases from 0° to 30°. At the system level, oblique incidence mainly shifts the reference output direction while preserving the lateral-translation-controlled steering relation. For the central MS I subaperture, the steering span over S_x = -300 to 300 μm increases only from 9.419° at normal incidence to 9.711° at an incidence angle of 20°, corresponding to a relative change of 3.10%. For the complete central row of subapertures, the overall angular span changes from 70.16° to 74.2°, corresponding to a relative change of 5.77% (Supplementary Figure S5a,c).

The illumination-coverage tuning shows a stronger dependence on the external incidence angle. The illumination coverage continues to increase monotonically with the axial-spacing change at all investigated incidence angles, indicating that the axial-spacing-controlled tuning mechanism remains effective. At the design spacing D_0, the residual angular spread increases from nearly zero under normal incidence to 0.839° at an incidence angle of 20°. At ΔD = 2.64 mm, the illumination coverage of the central

region increases from 9.554° under normal incidence to 12.501° at 20° incidence (Supplementary Figure S5b,c). The larger variation in illumination coverage results from the additional transverse displacement accumulated during interlayer propagation, which changes the local region sampled on the quadratic phase profile of MS II.

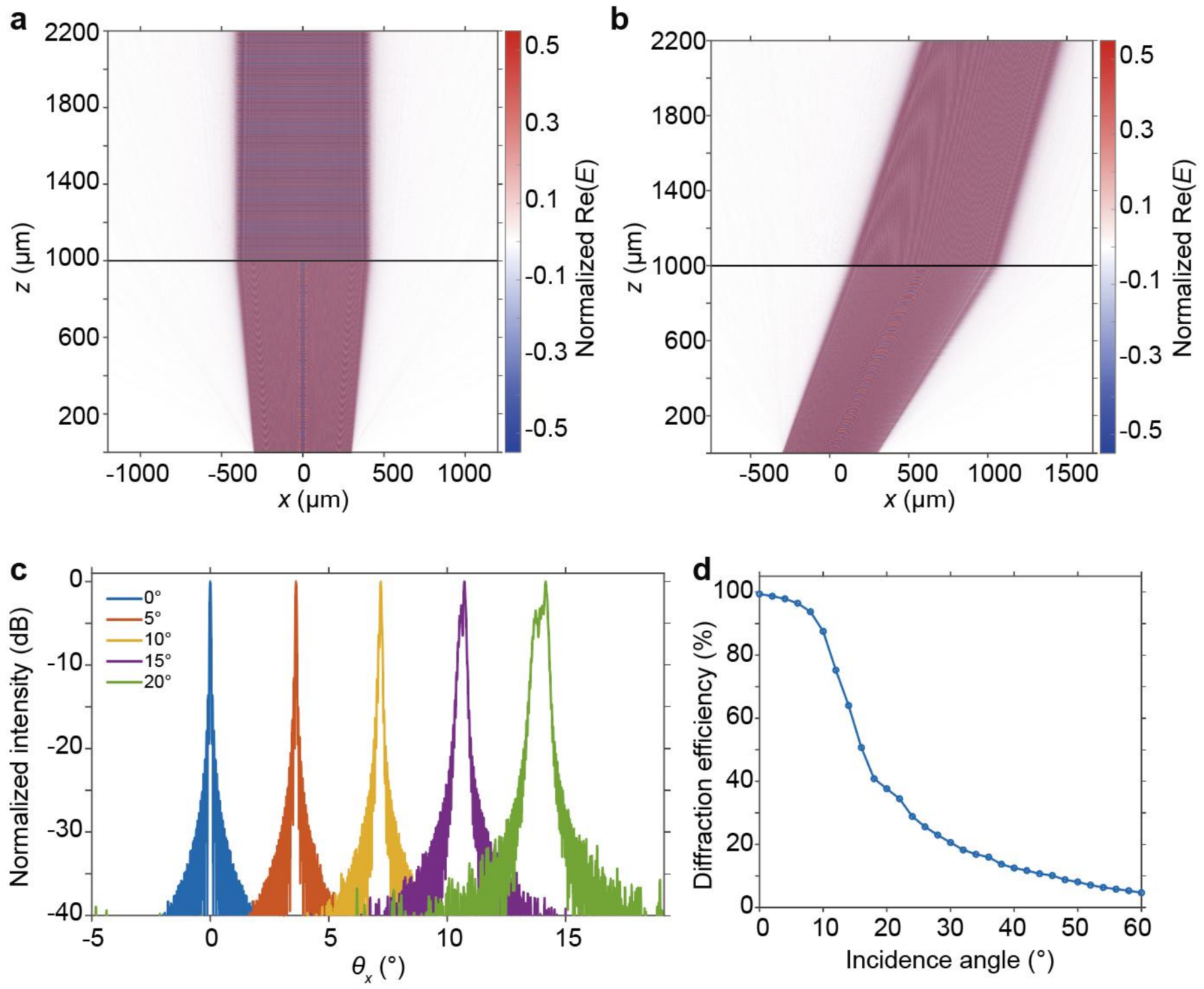

Supplementary Figure S6 | Oblique-incidence performance of the TATCMs. a, *x–z* propagation distribution under normal incidence. **b**, *x–z* propagation distribution under oblique incidence, showing the lateral displacement between MS I and MS II and the shifted output beam after MS II. **c**, Far-field angular spectra under external incidence angles from 0° to 20°, showing the progressive change in beam direction and FWHM. **d**, Designed-mode diffraction efficiency as a function of the external incidence angle.

We further evaluate the output beam quality using wave-optics simulations. The far-field output peak remains well defined as the external incidence angle increases from 0° to 20°, although the beam gradually broadens at larger incidence angles. The simulated FWHM is approximately 0.056° at both 0° and 5°, increases slightly to 0.065° at 10°, and reaches 0.098° and 0.142° at 15° and 20°, respectively (Supplementary Figure S6a–c). The designed-mode diffraction efficiency shows a similar trend. It remains relatively stable for small incidence angles and decreases more rapidly at larger angles because of increasing wavefront mismatch and redistribution of optical power away from the designed output mode (Supplementary Figure S6d).

These results show that external oblique incidence does not change the basic beam-steering and illumination-coverage tuning mechanisms of the TATCMs. The lateral beam-steering response remains relatively stable over the investigated angular range, whereas the illumination coverage and output beam quality become progressively more sensitive at larger incidence angles.

Supplementary Note 5: Illumination coverage at larger interlayer spacings

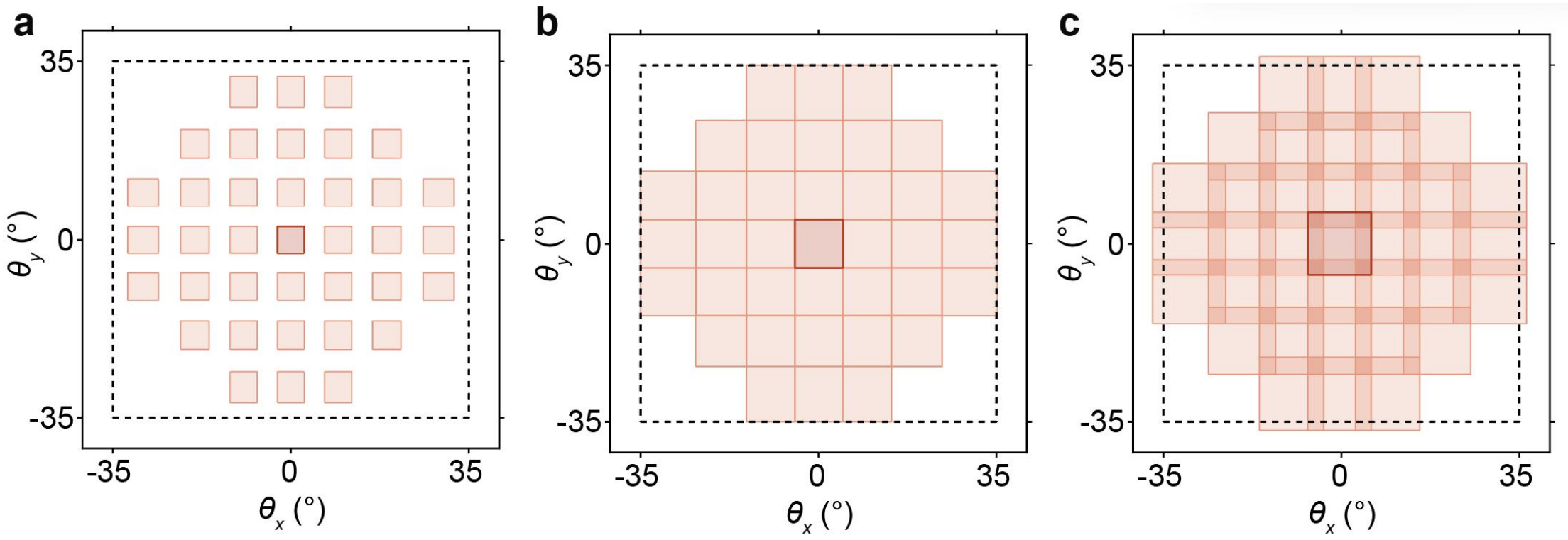

Supplementary Figure S7 | Illumination coverage at different interlayer spacings. Calculated illumination coverage at *S* = (0, 0) for a, D = 2.50 mm, **b,** D = 3.64 mm, **and c,** D = 4.50 mm.

The illumination coverage of the TATCMs continues to increase when the interlayer spacing exceeds the demonstrated value of D = 3.64 mm. We therefore further calculate the illumination coverage at larger interlayer spacings based on the relations derived in Supplementary Note 1. At D = 2.50 mm, the angular coverage generated by the central MS I subaperture is approximately 5.37°, and the overall illumination coverage is approximately 65.3° × 65.3°. Neighboring illumination regions remain separated at this spacing. At D = 3.64 mm, the angular coverage of the central MS I subaperture increases to approximately 9.45°, while the overall illumination coverage reaches approximately 70.2°×70.2°. Neighboring illumination regions become connected at this spacing and provide continuous coverage of the designed 70°×70° FoV. When D is further increased to 4.50 mm, the angular coverage of the central MS I subaperture increases to approximately 12.54°, and the overall illumination coverage expands to approximately 74.0°×74.0°. Neighboring illumination regions consequently overlap and extend beyond the designed FoV.

The selected value of D = 3.64 mm therefore does not represent the maximum interlayer spacing allowed by the theoretical model. We selected this spacing because it is sufficient to provide continuous coverage of the designed FoV in a single projection. Further increasing D mainly increases the overlap between neighboring illumination regions and extends the illumination beyond the target FoV. The larger illumination coverage also distributes the transmitted optical power over a wider angular region and reduces the local optical power density, which weakens the return signal from small or distant targets.

Supplementary Note 6: Scaling relation between illumination coverage and angular sampling efficiency

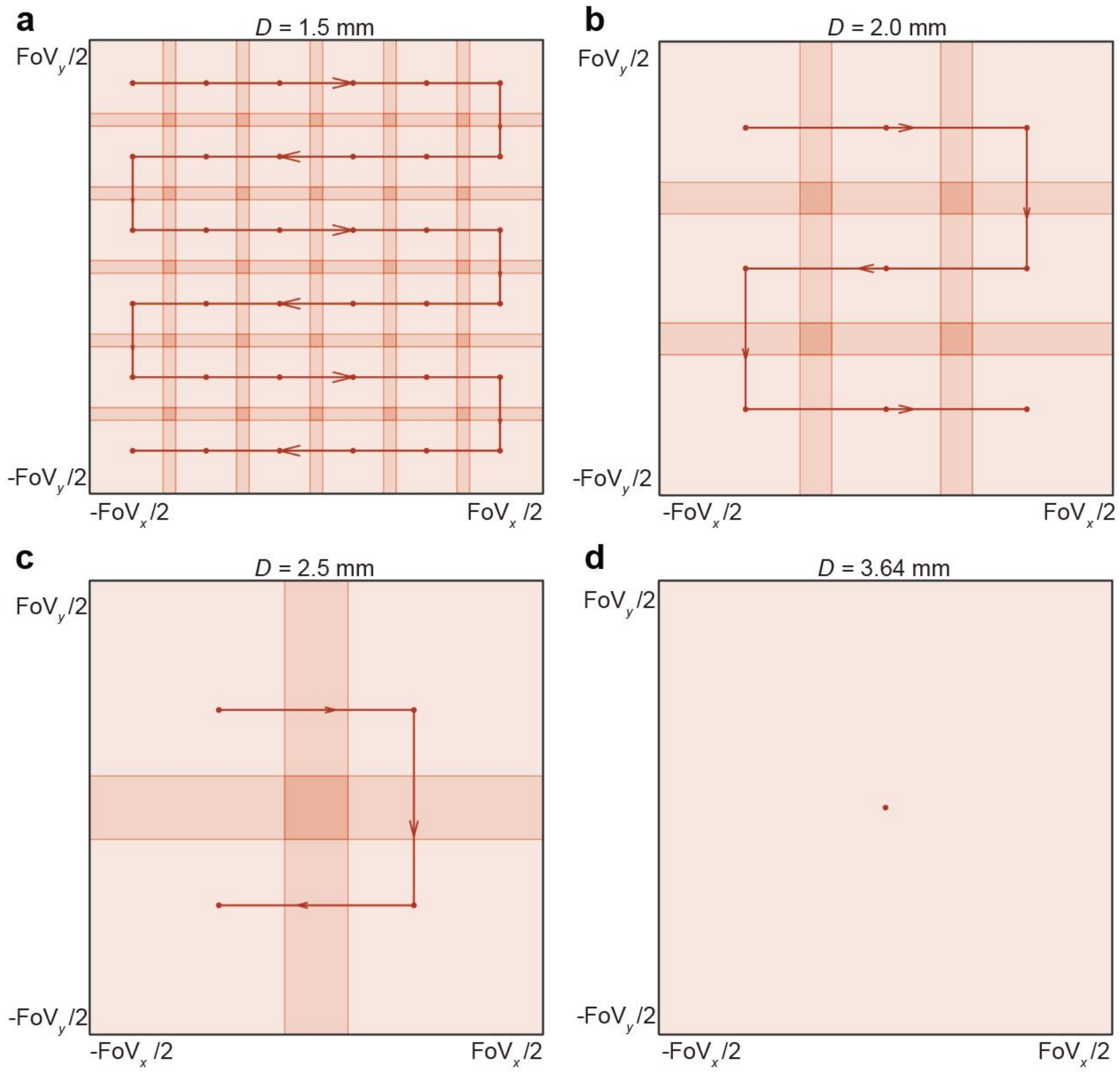

Supplementary Figure S8 | Angular scanning trajectories within the FoV subregion associated with each MS I subaperture under different illumination coverages.

This note establishes the scaling relation between illumination coverage and angular sampling efficiency within the FoV subregion associated with each MS I subaperture. Without loss of generality, we derive the relation between illumination coverage and angular sampling efficiency for the central illumination region. We define the normalized angular sampling efficiency as

$$\eta = \frac{1}{N_s} \tag{S.16}$$

Here, N_s is the minimum number of angular samples required to cover the corresponding FoV subregion. A value of $\eta = 1$ indicates that one angular sample covers the entire FoV subregion.

For the central illumination region, the lateral half-stroke of MS II determines the full output-angle span of the corresponding FoV subregion. Equation (3) in the main text gives the span along $q \in \{x, y\}$ as

$$\mathrm{FoV}_q = 2\arcsin\left(\frac{2|p|S_{\max}}{k_0}\right) \tag{S.17}$$

where $S_{\max}$ is the lateral half-stroke of MS II along the q direction. The symmetric

design gives $FoV_x = FoV_y$. For the TATCMs in this work, the full output-angle span of the central illumination region increases nearly linearly with $|\Delta D|$ over the axial tuning range. The full span along q is expressed as

$$\Omega_q(\Delta D) = \Omega_q^+(\Delta D) + \Omega_q^-(\Delta D) \approx \alpha_q|\Delta D| \tag{S.18}$$

where α_q denotes the increase in the full output-angle span per unit axial-spacing change along the q direction. Equation (S.18) neglects the small residual beam divergence at $D = D_0$.

The lateral translation of MS II samples discrete beam output angles within the FoV subregion assigned to the central MS I subaperture. The angular samples follow a serpentine trajectory, as illustrated in Supplementary Figure S8. The symmetry between the two transverse directions gives the minimum number of angular samples as

$$N_s = \left\lceil \frac{\mathrm{FoV}_q}{\Omega_q(\Delta D)} \right\rceil^2 \tag{S.19}$$

Substitution of Equations (S.18) and (S.19) into Equation (S.16) gives

$$\eta \approx \left[\frac{\Omega_q(\Delta D)}{\mathrm{FoV}_q}\right]^2 \propto |\Delta D|^2 \tag{S.20}$$

Equation (S.20) gives the scaling relation used in the main text. Increasing $|\Delta D|$ enlarges the illumination coverage associated with each angular sample, reduces the number of angular samples required to cover the FoV subregion and improves angular sampling efficiency.

Supplementary Note 7: Scaling relation between illumination coverage and maximum detectable distance

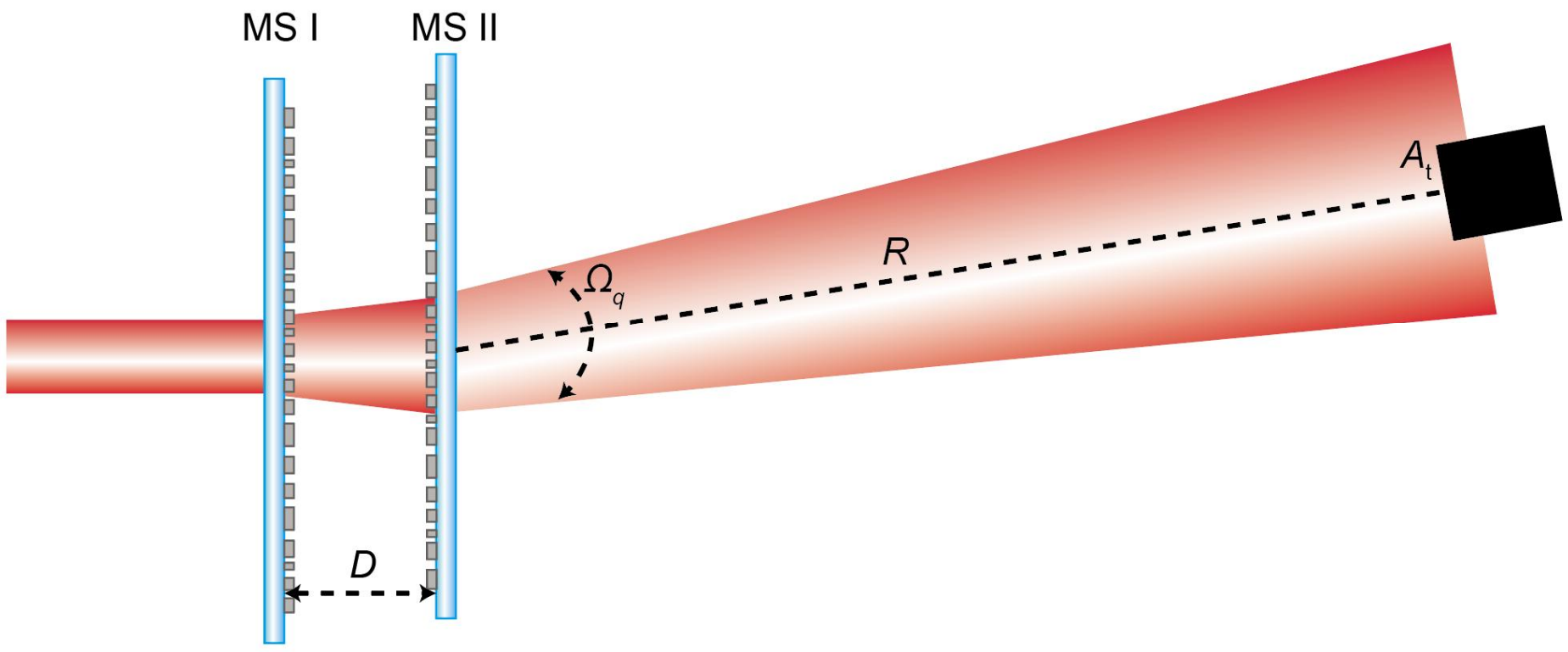

Supplementary Figure S9 | Schematic of beam expansion and optical-power collection from a finite target.

In active three-dimensional sensing, the maximum detectable distance is the farthest sensing distance at which the received optical power remains above the detection threshold of the receiver. The maximum detectable distance therefore reflects the ability of an illumination state to obtain sufficient return signals from distant or weak-return targets. Beam expansion reduces the optical power incident on the effective target area and consequently lowers the received optical power. We derive the scaling relation between illumination coverage and maximum detectable distance under this condition.

For a target located at a distance R from the TATCMs, the expanded illumination forms an approximately square illuminated area on the target plane. The area is given by $4R^2\tan^2[\Omega_q(\Delta D)/2]$. Assuming a uniform irradiance distribution over the illuminated area, the irradiance on the target plane is

$$E_t(R, \Delta D) = \frac{P_t}{4R^2\tan^2\left[\Omega_q(\Delta D)/2\right]} \tag{S.21}$$

where P_t is the optical power transmitted by the TATCMs and E_t is the irradiance on the target plane.

We consider the regime in which the illuminated area exceeds the effective target area A_t, as may occur for small targets or at long propagation distances. The optical power incident on the effective target area is therefore $E_t(R, \Delta D)A_t$. The receiver is positioned close to the TATCMs relative to the target distance, so the illumination and return propagation distances are both approximated by R. Assuming Lambertian reflection, the received optical power is

$$P_r(R, \Delta D) = \frac{P_t \eta_r \rho A_t A_r}{4\pi R^4\tan^2\left[\Omega_q(\Delta D)/2\right]} \tag{S.22}$$

where ρ is the target reflectivity, η_r is the receiving efficiency, A_r is the receiver aperture

area, and P_r is the received optical power.

For a fixed minimum received optical power $P_{r,\min}$, Equation (S.22) gives the maximum detectable distance as

$$R_{\max}(\Delta D) = \sqrt[4]{\frac{P_t \eta_r \rho A_t A_r}{4\pi P_{r,\min} \tan^2[\Omega_q(\Delta D)/2]}} \quad \text{(S.23)}$$

Within the angular range considered here, the tangent term in Equation (S.23) varies approximately linearly with the illumination coverage. The illumination coverage also increases nearly linearly with the axial-spacing change. Equation (S.23) therefore gives

$$R_{\max}(\Delta D) \propto |\Delta D|^{-1/2} \quad \text{(S.24)}$$

Equation (S.24) gives the scaling relation used in the main text. Expanded beam illumination increases illumination coverage but reduces the maximum detectable distance when the illuminated area exceeds the effective target area. Collimated beam illumination at $D = D_0$ concentrates more optical power on the target and therefore supports a longer maximum detectable distance.

Supplementary Note 8: Quantitative comparison of three illumination strategies

Following Supplementary Notes 6 and 7, we use $\Delta D = D - D_0$, D_0 = 1.00 mm, $q \in \{x, y\}$, $\Omega_q(\Delta D)$ and FoV_q. For a local square region, the discrete scan count N_s and efficiency η follow Equations (S.16) and (S.19). At D_{exp} = 3.64 mm (ΔD_{exp} = 2.64 mm), let $\Omega_{q,\mathrm{exp}}$ denote the expanded-state width. The continuous angular coverage ratio is

$$\eta_s(\Delta D) = \frac{\tan^2\left[\frac{\Omega_q(\Delta D)}{2}\right]}{\tan^2\left[\frac{\Omega_{q,\mathrm{exp}}}{2}\right]} \tag{S.25}$$

With common P_t, subaperture allocation and detection conditions, let $G_P(\Delta D)$denotes the return signal ratio:

$$G_P(\Delta D) = \frac{P_r(R, \Delta D)}{P_r(R, \Delta D_{\mathrm{exp}})} = \frac{1}{\eta_s(\Delta D)} \tag{S.26}$$

The detection range ratio can be derived from Equation (S.23). According to the above equations, we compare different indicators under three illumination states.

Supplementary Table S1 | Comparison of the three illumination states.

Illumination state	D(mm)	η_s	N_s	G_P	Range ratio
Fixed narrow beam	1.00	4.013×10^{-5}	24,964	24,919	12.564
TATCM refinement	1.60	0.05165	25	19.36	2.098
Fixed expanded	3.64	1	1	1	1

We also provide two numerical comparison figures to further visualize the benefit of our method. Supplementary Figure S10 illustrates the coverage-range trade-off of three illumination strategies. From left to right, narrow-beam scanning gives the strongest modeled return and smallest coverage, TATCM offers intermediate states, and fixed expanded illumination gives maximum coverage. The 0.060° FWHM supplies an equivalent finite width; the zero residual divergence approximation is not used at this endpoint. The model assumes uniform illumination, Lambertian reflection, negligible attenuation and a fixed target area smaller than every footprint. The range scaling need not hold when the beam fits inside the target or the effective sampled area changes with distance.

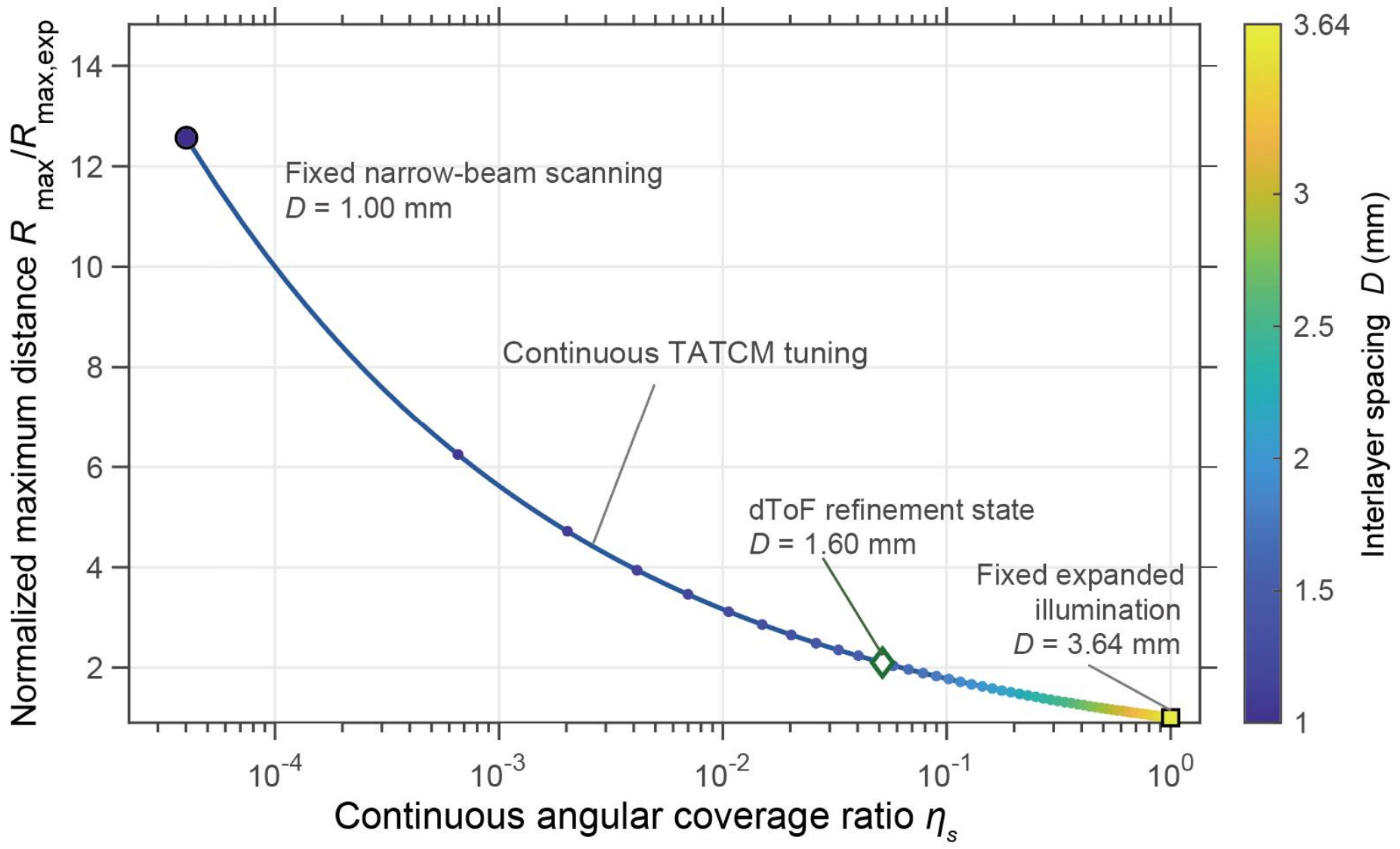

Supplementary Figure S10 | Coverage–range trade-off between angular coverage and detection range

For scene selection, define $R_{\mathrm{ref}} = R_{\mathrm{max,exp}}(\rho_{\mathrm{ref}})$ at reference reflectivity ρ_{ref} and $\eta_{\mathrm{s,narrow}} = \eta_s(0)$ for the finite narrow-state width. With other conditions fixed, the threshold and coverage-maximizing feasible value are:

$$\eta_s(\Delta D) \leq \frac{\frac{\rho}{\rho_{\mathrm{ref}}}}{\left(\frac{R}{R_{\mathrm{ref}}}\right)^4} \tag{S.27}$$

$$\eta_s{}^* = \min\left(1, \frac{\frac{\rho}{\rho_{\mathrm{ref}}}}{\left(\frac{R}{R_{\mathrm{ref}}}\right)^4}\right) \tag{S.28}$$

In Supplementary Figure S11, the expanded-state boundary is defined by:

$$\frac{\rho}{\rho_{\mathrm{ref}}} = \left(\frac{R}{R_{\mathrm{ref}}}\right)^4 \tag{S.29}$$

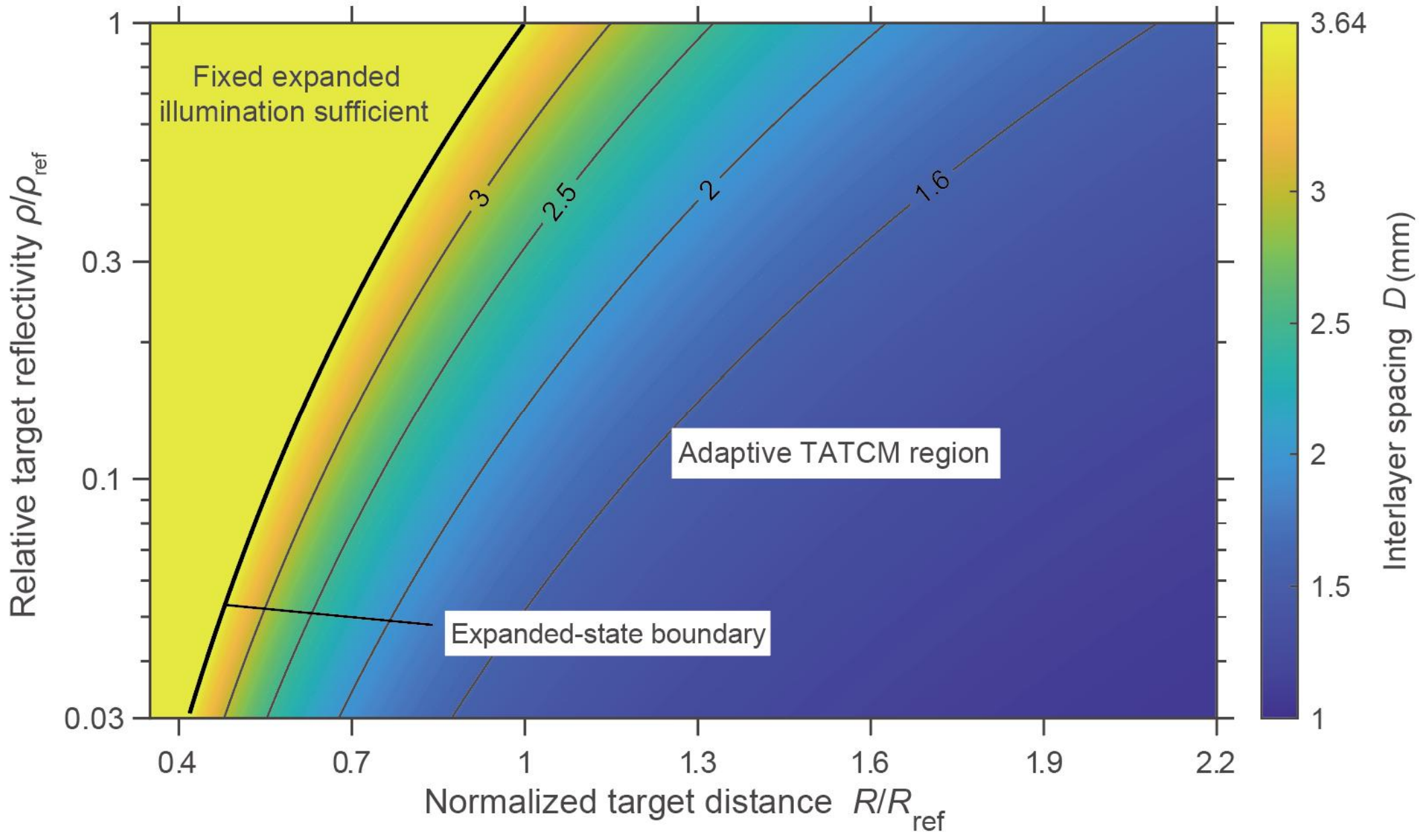

Supplementary Figure S11 | Scene-dependent selection of the interlayer spacing.

Lower reflectivity or greater distance requires narrower coverage. All points in the displayed map remain within the modeled narrow-beam detection envelope. Thus, the proposed benefit is the ability to select a suitable position along the coverage–signal trade-off for each scene.

Supplementary Note 9: Calibration and adaptive point-cloud reconstruction for the dToF sensing demonstration

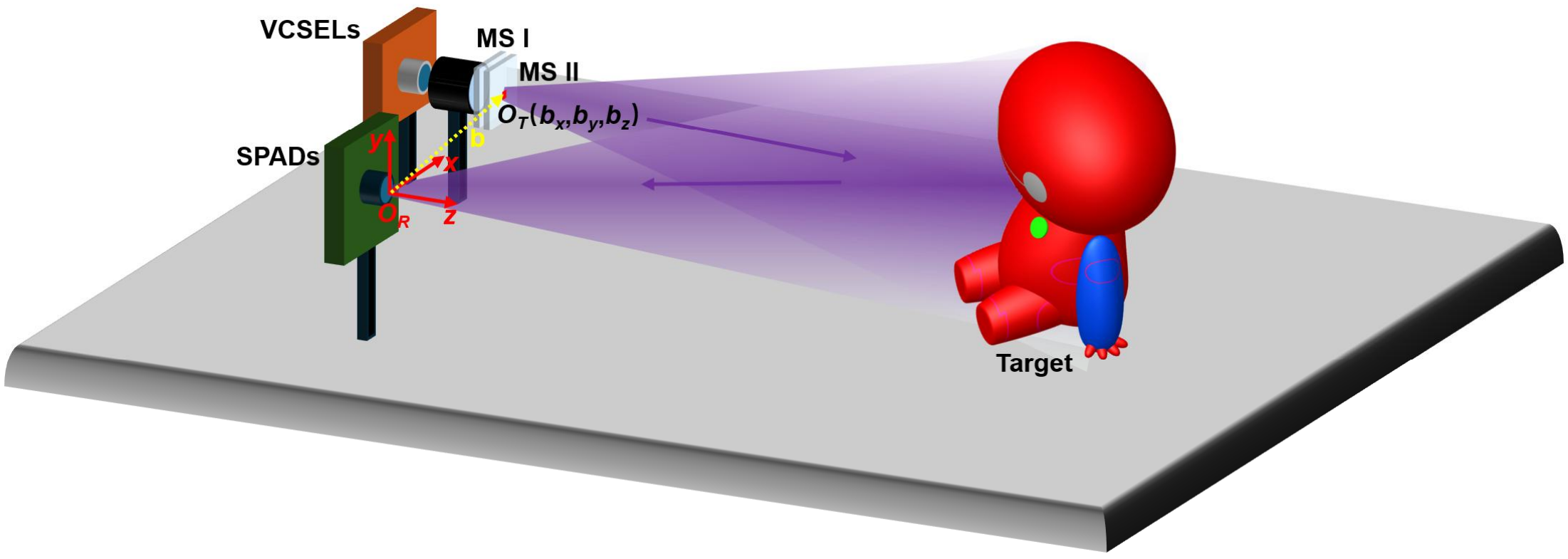

Supplementary Figure S12 | Experimental setup and coordinate systems of the dToF sensing system.

Supplementary Figure S12 shows the experimental setup and coordinate systems used for point-cloud calibration and reconstruction. The center of the SPAD receiver entrance pupil, O_R, defines the origin of the receiver coordinate system R. The TATCMs coordinate system T is centered at O_T, the center of the MS II aperture on the output plane. Because the target distances are much larger than the lateral size of the TATCM aperture, the output aperture is approximated as a point transmitter located at O_T. The baseline vector $\boldsymbol{b} = (b_x, b_y, b_z)$ points from O_R to O_T and is expressed in R. For each valid measurement, the commercial dToF module provides a processed three-dimensional point $\tilde{\boldsymbol{P}}_i = (\tilde{x}_i, \tilde{y}_i, \tilde{z}_i)$ and its return intensity. The reported point is decomposed into the radial distance $\tilde{\rho}_i = \|\tilde{\boldsymbol{P}}_i\|$ and the unit viewing direction $\tilde{\boldsymbol{d}}_i = \tilde{\boldsymbol{P}}_i/\tilde{\rho}_i$, which points from O_R toward the measured target point.

The commercial dToF module reconstructs target points using its internal calibration for the relative positions and optical paths of its original built-in transmitter and receiver. In our system, the emitted light is routed through the external collimation optics and TATCMs, so the center of the MS II output aperture replaces the original built-in transmitter as the effective emission position, while the receiver optics remain unchanged. The unit viewing direction $\tilde{\boldsymbol{d}}_i$, which is determined by the receiver pixel-to-angle calibration, is therefore retained. The reported radial distance $\tilde{\rho}_i$ is recalibrated to account for the distance-scale error, the additional fixed optical path introduced by the external transmitter optics, and the spatial separation between the transmitter and receiver. The corrected total propagation length from the effective transmitter O_T to the target and then to the receiver O_R is defined as $L_i = 2(\alpha_D\tilde{\rho}_i + \beta_D)$. The corrected receiver-to-target distance is then

$$\rho_i = \frac{L_i^2 - \|\boldsymbol{b}\|^2}{2\left(L_i - \boldsymbol{b}^{\mathrm{T}}\tilde{\boldsymbol{d}}_i\right)} \tag{S.30}$$

Here, α_D compensates for the proportional error in the distance reported by the dToF module, and β_D accounts for the constant additional optical path introduced by the external transmitter optics. Retaining the measured viewing direction $\tilde{\boldsymbol{d}}_i$, the corrected target position in R is $\boldsymbol{P}_{R,i} = \rho_i \tilde{\boldsymbol{d}}_i$. To combine measurements acquired under different illumination states, the corrected point $\boldsymbol{P}_{R,i}$ is transformed from the receiver coordinate system R to the experimental reference coordinate system W according to

$$\boldsymbol{P}_{W,i} = \boldsymbol{R}_{WR}\boldsymbol{P}_{R,i} + \boldsymbol{t}_{WR} \tag{S.31}$$

where $\boldsymbol{R}_{WR}$ and $\boldsymbol{t}_{WR}$ are the rotation matrix and translation vector from R to W, respectively.

The geometric and distance-calibration parameters were jointly determined using a planar target placed at known distances and positions across the working range and FoV. Calibration measurements were acquired at both interlayer spacings used in the sensing experiment. Mechanical measurements provided an initial estimate of the transmitter–receiver baseline, which was subsequently refined together with the rigid transformation from R to W and the distance-calibration coefficients α_D and β_D for each interlayer spacing. The parameters were fitted by minimizing the distances between the corrected points and the known reference planes, while reducing the influence of outliers. Return intensity was used only to reject invalid measurements and reduce the contribution of weak returns. It was not used to modify the measured depth. Calibration accuracy was evaluated using separate planar measurements that were not included in the parameter fitting.

After calibrating the distance measurements and the transmitter–receiver geometry, we determined the relative orientation between the TATCM coordinate system T and the receiver coordinate system R, together with the stage position corresponding to $\boldsymbol{S}$ = (0, 0). At the design spacing D_0, MS II was translated to a series of known positions, and the corresponding illumination centers on a planar target were extracted from the return-intensity data. For the i-th target point, the transmitter-to-target direction expressed in T is

$$\boldsymbol{d}_{Tx,i}^{T} = \boldsymbol{R}_{RT}^{T} \frac{\boldsymbol{P}_{R,i} - \boldsymbol{b}}{||\boldsymbol{P}_{R,i} - \boldsymbol{b}||} \tag{S.32}$$

Here, $\boldsymbol{R}_{RT}$ is the rotation matrix from T to R. The relative rotation $\boldsymbol{R}_{RT}$ was determined together with the stage position corresponding to $\boldsymbol{S}$ = (0, 0) by fitting the measured illumination directions at known MS II translations to Equation (3) in the main text. The required MS II displacement along $q \in \{x, y\}$ is

$$S_{q,i} = c_{q,nm} - \frac{k_0}{2p} d_{Tx,q,i}^{T} \tag{S.33}$$

Here, $S_{q,i}$ is the MS II translation required to illuminate the i-th target point, $c_{q,nm}$ is the q-coordinate of the selected MS I subaperture center, and $d_{Tx,q,i}^{T}$ is the q-direction component of the target direction expressed in T.

The calibration parameters were applied to the initial measurements acquired under expanded illumination and the subsequent measurements acquired by scanning with reduced illumination coverage. The resulting point clouds were transformed into the common coordinate system *W*. The initial point cloud was acquired at D = 3.64 mm under expanded illumination to reconstruct scene regions that produced valid returns. A measured point was retained only when its three reported coordinates were finite and its return intensity exceeded a preset threshold. In this proof-of-concept experiment, the angular region containing the distant black cube was predefined for subsequent scanning. Because the initial point cloud contained no valid returns from this region, the interlayer spacing was reduced and the region was rescanned with reduced illumination coverage and increased optical-power concentration. This experiment demonstrates illumination-state adaptation for a predefined region with insufficient returns rather than autonomous target detection.

For the subsequent scanning measurements, the interlayer spacing was reduced to D = 1.60 mm to decrease the angular coverage of each projected illumination region and increase the optical-power concentration. Based on the sampling criterion in Equation (S.19), five projection centers were used along each transverse direction, resulting in a 5×5 serpentine scanning trajectory comprising 25 projection positions. The measurements acquired under expanded illumination and reduced-coverage scanning were corrected using the calibration parameters corresponding to their respective interlayer spacings. At each scanning position, measured points were retained only within the angular coverage of the corresponding projected illumination region. The two point clouds were then combined by voxel averaging in the common coordinate system. The initial point cloud provided the scene regions reconstructed under expanded illumination, while the subsequent scanning measurements reconstructed the previously missing surface of the distant cube without surface completion. The depth accuracy of the reconstructed black cube was evaluated using its four visible vertices. For $l \in \{1, 2, 3, 4\}$, z_l and z_l^{ref} denote the reconstructed and independently measured reference depths of vertex l in *W*, respectively. The relative depth error of each vertex is

$$\varepsilon_{z,l} = \frac{\left|z_l - z_l^{\mathrm{ref}}\right|}{z_l^{\mathrm{ref}}} \times 100\% \tag{S.34}$$

where $\varepsilon_{z,l}$ is the percentage depth error of vertex l. The mean relative depth error over the four vertices is

$$\bar{\varepsilon}_z = \frac{1}{4}\sum_{l=1}^{4} \varepsilon_{z,l} \tag{S.35}$$

Spatial depth errors were further evaluated over the reconstructed scene containing the elephant and Baymax models and two nominal planar regions. The point cloud was obtained by merging the flash acquisition with the scan acquisitions described in the preceding reconstruction procedure. Supplementary Figure S13 presents the spatial distribution of relative depth errors across the evaluated FoV.

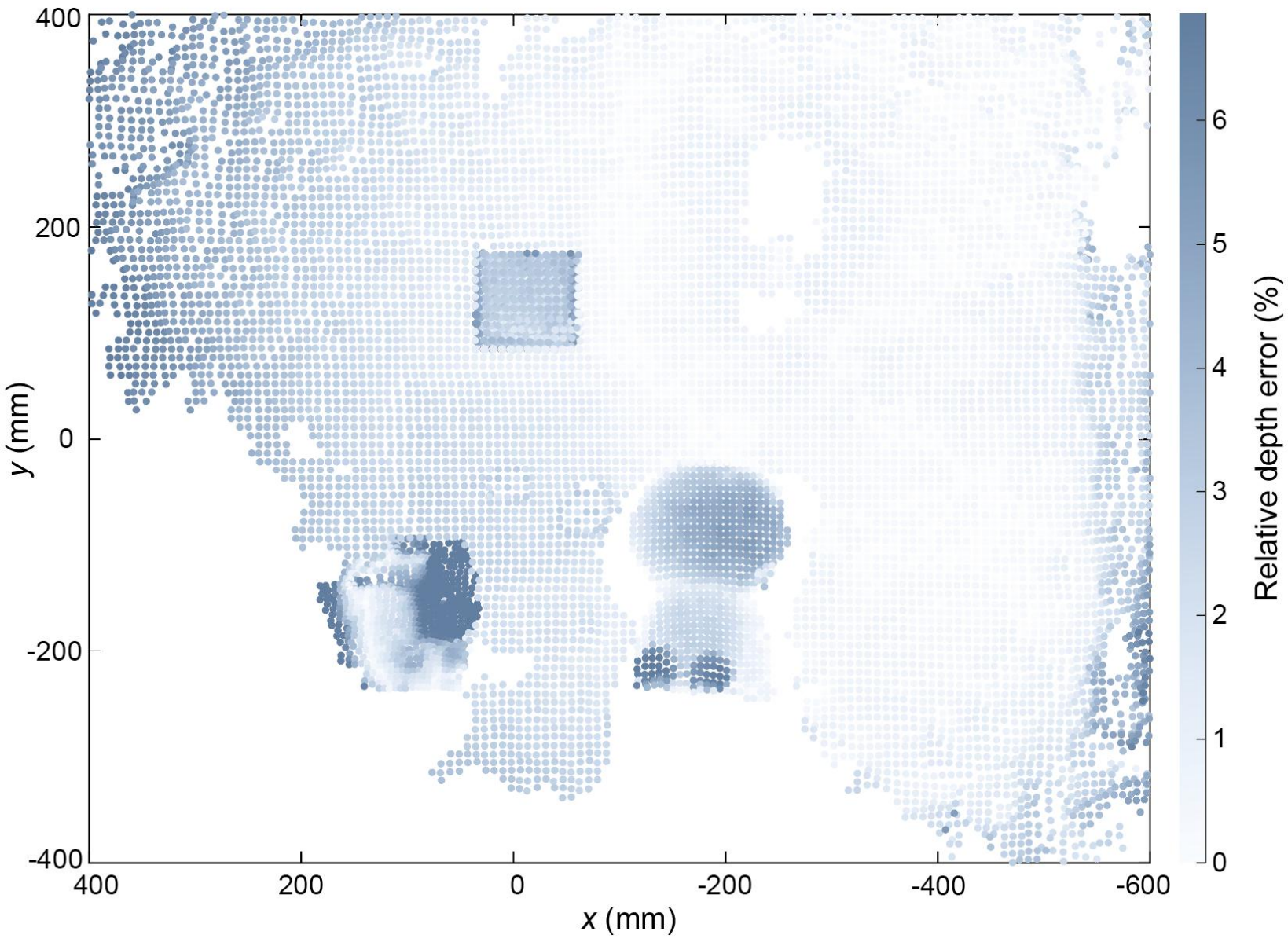

Supplementary Figure S13 | Spatial distribution of relative depth errors across the reconstructed scene

For the elephant and Baymax objects, the measured point clouds were aligned with their CAD models using coarse pose initialization followed by iterative closest-point (ICP) refinement, with the resulting transformations fixed during evaluation. Reference depths were obtained from the corresponding visible CAD surfaces near each valid (x,y) location. Unsupported or ambiguous correspondences, particularly near silhouettes and steep surfaces, were excluded. The two planar regions were assigned nominal reference depths from their measured positions in the common coordinate system W, using nonoverlapping masks that excluded foreground objects.

Supplementary Figure S13 shows lower relative depth errors over much of the central background and higher values in parts of the periphery and localized object regions. Spatial variations in return quality and residual calibration errors may contribute to this pattern. Near object boundaries and steep surfaces, small lateral alignment errors can produce larger depth residuals, while departures of the physical surfaces from their nominal planar or CAD references may also contribute. These relative errors therefore provide a spatially resolved, reference-based assessment of reconstruction consistency and should not be interpreted as independent pointwise measurements of absolute ranging accuracy.

Supplementary Note 10: Prospective framework for adaptive illumination

Building on the demonstrated reconstruction capabilities, we next consider how illumination control could be integrated with sensing feedback and decision-making to support autonomous scene-dependent adaptation. Supplementary Figure S14 illustrates a prospective closed-loop framework for scene-dependent adaptive illumination based on the illumination-control and 3D reconstruction capabilities demonstrated in this work. The TATCM provides two key degrees of optical control: adjustment of the interlayer spacing D modifies the illumination coverage and local power concentration, while lateral translation of MS II changes the output-beam directions and enables spatial rescanning. These controllable illumination states, together with the demonstrated depth acquisition, registration, and point-cloud fusion procedures, provide the physical basis for adaptive operation.

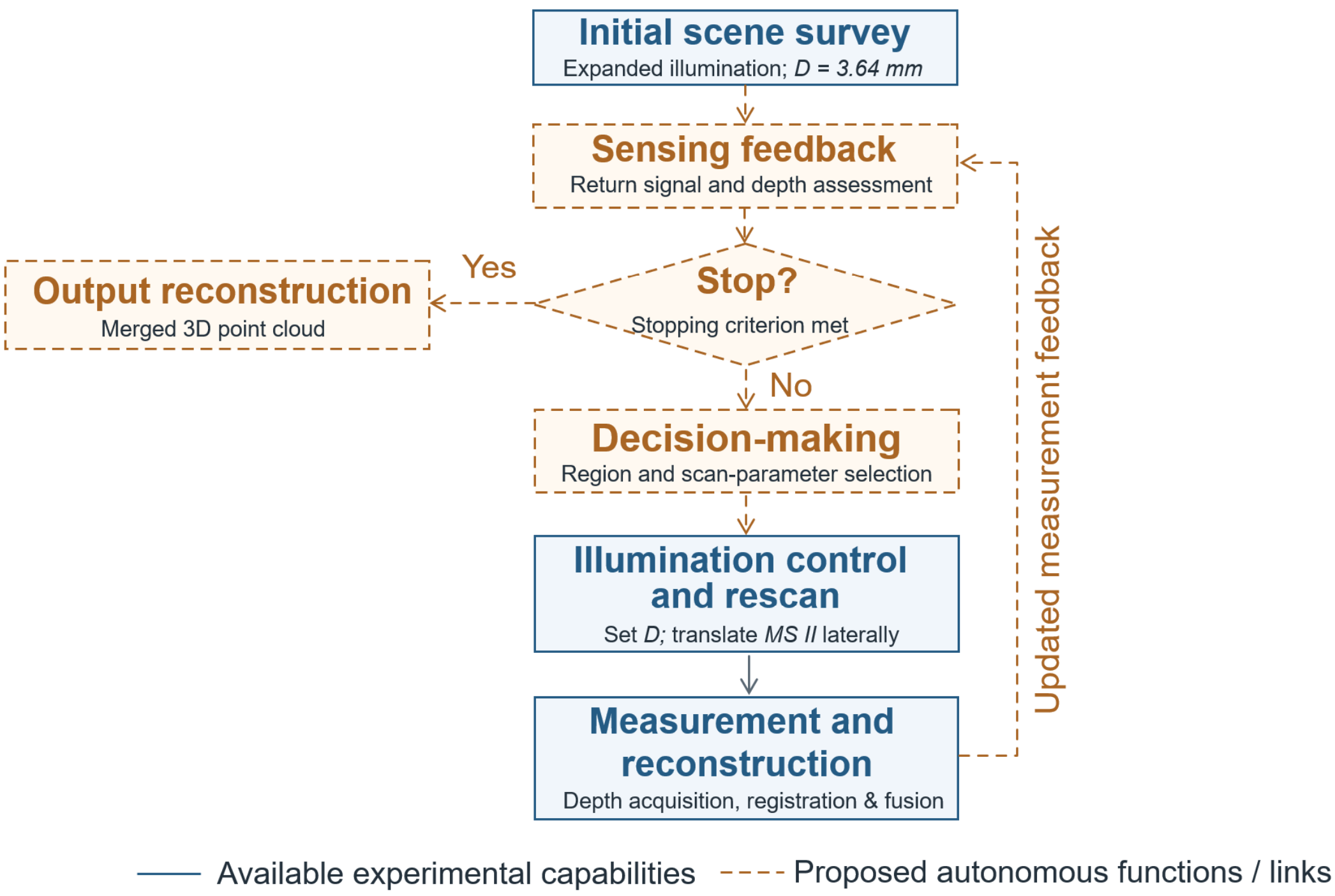

Supplementary Figure S14 | Prospective closed-loop framework for autonomous scene-dependent adaptive illumination.

In a prospective autonomous implementation, an initial scene survey would first be performed using expanded illumination, such as the D = 3.64 mm state used in the present experiment. The returned signal and reconstructed depth information would then be evaluated to identify regions requiring further measurement. Available quantities such as return strength, measurement confidence, or repeated-measurement consistency could serve as online indicators of measurement quality. Regions with sufficiently reliable measurements would be retained, whereas regions with detectable

but inadequate returns could be selected for enhanced illumination. Importantly, the controller would not have access to the true depth error of an unknown scene, and a missing depth measurement alone would not distinguish empty space from a weakly reflecting or otherwise unresolved target.

Based on this sensing feedback, a decision module could determine whether additional acquisition is required and, if so, select the region of interest and corresponding scan parameters. The illumination state could then be reconfigured by adjusting D to modify local illumination concentration and translating MS II laterally to scan the selected region. The relationship between these mechanical settings, beam direction, and illumination coverage can be established through system calibration. Because the MS I subaperture beams share the same mechanical degrees of freedom, their illumination footprints would be considered jointly rather than controlled independently. Newly acquired depth data would subsequently be registered and fused with the existing reconstruction, after which the updated measurements would be reassessed through the same feedback loop.

The iteration would terminate when predefined task-specific quality criteria are satisfied, the available acquisition budget is reached, or no further useful measurement action remains, yielding the merged 3D point cloud as the final output. The present laboratory experiment establishes the key reconfigurable illumination and reconstruction capabilities required by this framework, while the sensing-feedback assessment, autonomous decision-making, and integrated closed-loop control shown in Supplementary Figure S14 represent a possible system-level extension. Their full implementation and validation will require further engineering of the feedback interface, decision criteria, and coordinated sensing–actuation control.

Supplementary Note 11: Comparison with representative beam-steering and mechanically actuated approaches

Different beam-steering approaches rely on different mechanisms for generating and dynamically reconfiguring the optical wavefront. Supplementary Table S2 compares representative LCOS-based beam steering, electrically tunable metasurfaces, mechanically translated cascaded metasurfaces, and the TATCMs demonstrated in this work. The comparison focuses on experimentally demonstrated steering range, dynamic-control mechanism, required control or actuation scale, and reported optical efficiency. SLMs and electrically tunable metasurfaces provide direct electronic control of the spatial phase distribution, whereas mechanically tunable cascaded metasurfaces encode the fine spatial phase modulation into passive metasurface profiles and reconfigure the output wavefront through relative motion between the phase planes. Compared with the previous dual-mode cascaded-metasurface approach[1], the present TATCMs further enable continuous tuning of the illumination coverage through axial-spacing control.

Supplementary Table S3 further compares representative mechanically actuated scanning approaches from the perspective of mechanical motion and dynamic response. For rotational scanners, the optical deflection is generated directly by angular motion of a mirror or other optical element, and the achievable frequency therefore depends strongly on the required scan angle, moving aperture, and inertia. Translational actuation exhibits an analogous stroke–frequency trade-off. Compared with the previous laterally translated cascaded metasurface[2], the present subaperture architecture reduces the required translation from the millimeter scale to the hundred-micrometer scale. Reported in-plane MEMS actuators have demonstrated hundred-micrometer-scale travel together with resonant frequencies ranging from approximately 100 Hz to several hundred hertz, although the reported travel definitions and operating conditions differ among actuator architectures. These results indicate the feasibility of substantially faster lateral translation than that provided by the motorized stages used in the present experiments. The motorized translation stages used in the present experiments were selected for quasi-static optical characterization and do not represent the dynamic limit of a future MEMS-integrated implementation.

Supplementary Table S2 | Comparison of representative reconfigurable beam-steering approaches.

Reference	Approach	Steering range	Control / actuation	Efficiency	Remarks
[3]	LCoS SLM	±15.6°, 1D	3.6 μm grating pixels	2.28% at max. angle	Large-angle LCoS diffraction steering
[4]	LCoS SLM	10°, 1D	Pixel-wise phase modulation	>37.8%	Fringe-field compensation
[5]	ITO metasurface	±7.3°, 2D	10 × 10 pixels, 5.2 μm pitch	3.4–6.9%	2D electrical addressing
[6]	LC metasurface	22°, 1D	96 electrodes, 1 μm pitch	~23% at max. FoV	Near-2π phase modulation
[7]	CMOS–LC metasurface	24°, 1D	2500 electrodes, 0.8 μm pitch	>18% at max. angle	Submicrometer electrical addressing
[1]	Hybrid cascaded metasurfaces	±35°, 2D	±100 μm translation; polarization switching	~20% scanning;	Two predesigned illumination modes
This work	TATCMs	±35°, 2D	±300 μm lateral + 2.64 mm axial tuning	64.8%	Continuous beam-direction and coverage control

Supplementary Table S3 | Comparison of representative mechanically actuated beam-steering approaches.

Reference	Approach	Mechanical motion	Optical scan range	Frequency	Remarks
[8]	Rotating LiDAR	Full-aperture rotation	360°	5–20 Hz	Full-aperture mechanical rotation
[9]	Galvanometer scanner	Polygon rotation and galvo rotation	27° × 20°	200 Hz	Hybrid fast- and slow-axis scanning
[10]	Electromagnetic MEMS mirror	Biaxial mirror rotation	36.3°×35.2°	132 / 712 Hz	7.2-mm mirror ; slow / fast axes
[11]	Piezoelectric MEMS mirror	Biaxial resonant rotation	102.8°×104.8°	810 / 891 Hz	5-mm mirror; vacuum operation
[12]	Electrostatic translational MEMS	0.18 mm translation	—	769 Hz	1D large-stroke actuator
[13]	Electromagnetic translational MEMS	±0.40 mm translation	—	96.7 Hz	2D large-stroke actuator
[2]	Cascaded metasurfaces	±3.5 mm translation	90°×90°	—	Transmissive; mm-scale translation
This work	TATCMs	±0.30 mm translation	70°×70°	—	Transmissive; sub-mm translation

References

1 Zhang, L. *et al.* A dual-mode LiDAR system enabled by mechanically tunable hybrid cascaded metasurfaces. *Light: Science & Applications* **14**, 287 (2025).

2 Zhang, L. *et al.* Highly tunable cascaded metasurfaces for continuous two-dimensional beam steering. *Advanced Science* **10**, 2300542 (2023).

3 Son, S.-J. *et al.* Wide optical beam steering LCOS device for solid-state LiDAR applications. *IEEE Photonics Technology Letters* **36**, 35-38 (2023).

4 Hu, Y. *et al.* Achieving high diffraction efficiency in LCoS-SLMs via neural-network-based precise fringe-field compensation. *Optics Express* **34**, 15877-15889 (2026).

5 Kim, S. I. *et al.* Two-dimensional beam steering with tunable metasurface in infrared regime. *Nanophotonics* **11**, 2719-2726 (2022).

6 Moitra, P. *et al.* Electrically tunable reflective metasurfaces with continuous and full-phase modulation for high-efficiency wavefront control at visible frequencies. *Acs Nano* **17**, 16952-16959 (2023).

7 Dong, C. *et al.* High-Efficiency Near-Infrared Beam Steering Enabled by a CMOS-Driven Liquid-Crystal Metasurface. *Nano Letters* **26**, 6341-6348 (2026).

8 Yang, D. *et al.* Development of the high angular resolution 360 LiDAR based on scanning MEMS mirror. *Scientific Reports* **13**, 1540 (2023).

9 Prasad, N. S. & Mylapore, A. R. High-speed wide-angle interleaved scanning technique for a 3D imaging lidar. *Journal of the Optical Society of America B* **38**, 22-27 (2021).

10 Fang, X.-Y. *et al.* A 2D MEMS crosstalk-free electromagnetic micromirror for LiDAR application. *Journal of Microelectromechanical Systems* **33**, 559-567 (2024).

11 Huang, H. *et al.* Mechanical robust biaxial MEMS mirror achieving> 100 optical scanning angle for LiDAR application. *IEEE Sensors Journal* **25**, 4306-4315 (2024).

12 Grade, J. D., Jerman, H. & Kenny, T. W. Design of large deflection electrostatic actuators. *Journal of Microelectromechanical Systems* **12**, 335-343 (2003).

13 Bian, W. *et al.* Design method for out-of-plane motion rejecting structure in 2-DoF large stroke actuators. *Microsystems & Nanoengineering* **11**, 144 (2025).
